# Soft like velvet and shiny like satin: Perceptual material signatures of fabrics depicted in 17^th^ century paintings

**DOI:** 10.1167/jov.21.5.10

**Published:** 2021-05-12

**Authors:** Francesca Di Cicco, Mitchell J. P. van Zuijlen, Maarten W. A. Wijntjes, Sylvia C. Pont

**Affiliations:** 1 Perceptual Intelligence Lab, Faculty of Industrial Design Engineering, Delft University of Technology, Delft, The Netherlands; 2 Perceptual Intelligence Lab, Faculty of Industrial Design Engineering, Delft University of Technology, Delft, The Netherlands; 3 Perceptual Intelligence Lab, Faculty of Industrial Design Engineering, Delft University of Technology, Delft, The Netherlands; 4 Perceptual Intelligence Lab, Faculty of Industrial Design Engineering, Delft University of Technology, Delft, The Netherlands

**Keywords:** material perception, fabrics, softness, shininess, image cues, paintings

## Abstract

Dutch 17^th^ century painters were masters in depicting materials and their properties in a convincing way. Here, we studied the perception of the material signatures and key image features of different depicted fabrics, like satin and velvet. We also tested whether the perception of fabrics depicted in paintings related to local or global cues, by cropping the stimuli. In Experiment 1, roughness, warmth, softness, heaviness, hairiness, and shininess were rated for the stimuli shown either full figure or cropped. In the full figure, all attributes except shininess were rated higher for velvet, whereas shininess was rated higher for satin. This distinction was less clear in the cropped condition, and some properties were perceived significantly different between the two conditions. In Experiment 2 we tested whether this difference was due to the choice of the cropped area. On the basis of the results of Experiment 1, shininess and softness were rated for multiple crops from each fabric. Most crops from the same fabric differed significantly in shininess, but not in softness perception. Perceived shininess correlated positively with the mean luminance of the crops and the highlights’ coverage. Experiment 1 showed that painted velvet and satin triggered distinct perceptions, indicative of robust material signatures of the two fabrics. The results of Experiment 2 suggest that the presence of local image cues affects the perception of optical properties like shininess, but not mechanical properties such as softness.

## Introduction

Fabrics serve a wide array of functions in our daily life. We use fabrics to hold and carry things, to clean and dry surfaces, for decoration, and for clothing. With this wide array of functions, the material category of “fabric” also comes with a wide variety of appearances. The visual appearance of fabrics depends on the type of fiber (e.g., natural or synthetic), the yarn (the continuous segment of fibers), and the weaving method (Koenderink & Pont, 2003; Pont & Koenderink, 2003; Zhao, Jakob, Marschner, & Bala, 2011). Materials’ appearances are strongly dependent on light (Pont & te Pas, 2006; Fleming, Dror, & Adelson, 2003) and shape (Ho, Landy, & Maloney, 2008; Marlow & Anderson, 2015; Schmidt, Fleming & Valsecchi, 2020). This is true also for the appearance of fabrics, which has been shown to depend on the illumination environment (Barati, Karana, Sekulovski, Pont, 2015; Zhang, de Ridder, Barla, & Pont, 2019) and on the folding shape (Xiao, Bi, Jia, Wei, & Adelson, 2016). Nonetheless, we can visually discriminate and identify different types of fabrics on the basis of their characteristic visual qualities, also known as “material signatures” (Fleming, Wiebel, & Gegenfurtner, 2013).

**Figure 1.**
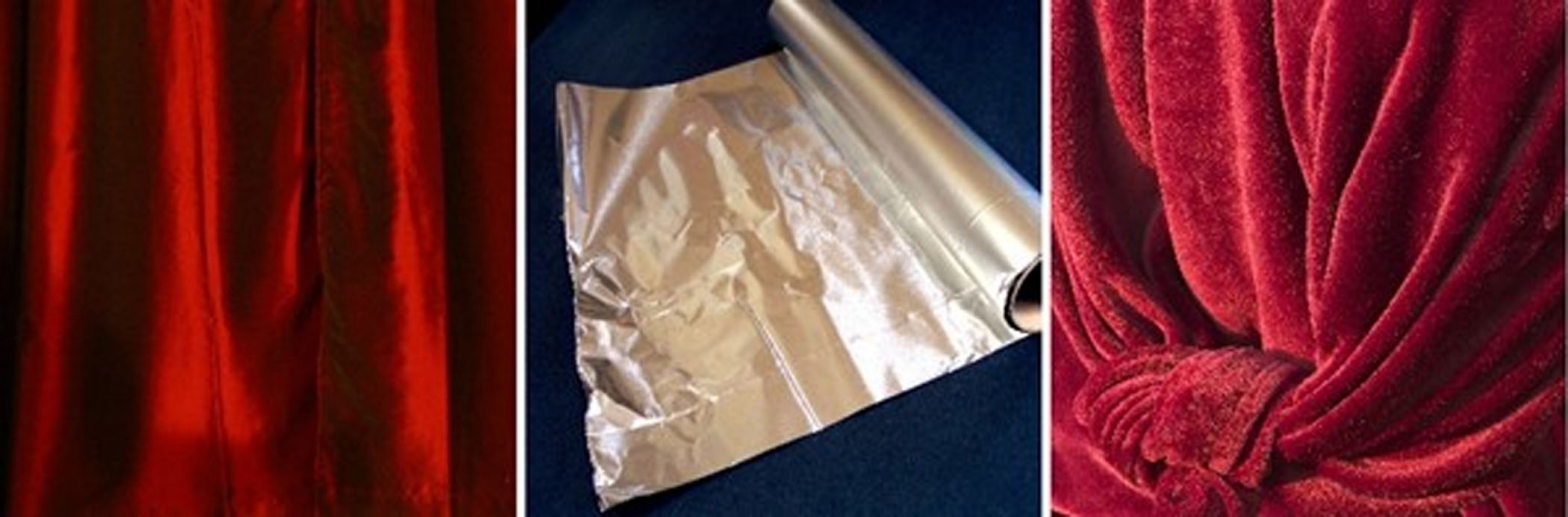
Satin (left) is visually more similar to aluminum foil (middle) than to velvet (right). However, satin certainly belongs to a different material class than aluminum. The first two images were downloaded from Morguefile.com and the third image from pxfuel.com, released under free license.

In this article we focus on the appearance of velvet and satin. Velvet and satin both belong to the material category of fabric, but large differences exist within this same material class. On visual observation, one could find more similarities between the appearance of satin and aluminum foil than between satin and velvet (Figure 1). However, despite the visual similarity, nobody would classify aluminum as a fabric.

In this study, we studied the perception of painted fabrics in 17^th^ century Dutch paintings, a class of paintings unanimously acknowledged for the convincing representation of materials and their properties. The economical yet effective rendering of material properties exploited by 17^th^ century painters (Parraman, 2014) resonates with the mechanisms of the human visual system (Adelson, 2001; Koenderink & van Doorn, 2001; Cavanagh, 2005; Sayim & Cavanagh, 2011; Wijntjes, Doerschner, Kucukoglu, & Pont, 2012; Marlow, Kim, & Anderson, 2017; Di Cicco, Wijntjes & Pont, 2019; Van Zuijlen, Pont, & Wijntjes, 2020; Wijntjes, Spoiala & de Ridder, 2020). Painters carefully chose the image features to include and could choose to omit perceptually irrelevant or hindering features, as was shown to be the case for the orientation of the highlights on grapes which do not need to be congruent with the object shape in order to communicate a glossy appearance (Di Cicco et al., 2019). Materials were often painted according to standard, well-established instructions which assured the painter of getting the best possible rendering. Velvet, for example, could be convincingly depicted by simply inverting the typical patterns of light and shade (Lu, Koenderink, & Kappers, 1998; Van Duijn & Roeders, 2012). Written records of such visual tricks can be found in *Het Schilder-boek*, a book describing the life and work of several painters, composed by Dutch painter and art historian Karel van Mander in 1604. He wrote: “In contrast to your other textile, where you render with light paint all the relief in the folds, this is completely different with velvet [drapery], as you make these entirely dark and paint flat highlights only on the reflecting side” (Van Mander, 1604; Van Eikema Hommes, 2002). Another relevant art historical source is “The Big World Painted Small” by Willem Beurs (1692, Lehmann & Stumpel, in press). This book has already proven to be a useful tool to help understand pictorial procedures and the relevant image features for the rendering of materials (Di Cicco, Wiersma, Wijntjes, & Pont, 2020). In this collection of pictorial recipes, Beurs described how to paint satin and velvet, emphasizing the different rendering of specular reflections, sharp and high contrast for satin and somewhat blurrier and with less contrast for velvet (Pottasch, 2020). These are examples of the value of investigating paintings and art historical writings for the sake of understanding the functioning of the human visual system.

Understanding the material attributes that form the signatures of the representation of different fabrics, like velvet and satin, is important for several applications. One example is online shopping, in which visual communication of the material qualities of fabrics is crucial to guide the consumers’ choice. The appearance in the image should match as closely as possible the appearance that would be perceived in a real shop. Failing to capture and convey the material attributes of the fabric is one of the major concerns of online retailing (Tuunainen & Rossi, 2002). On this topic, it has been shown that dynamic stimuli (videos) can better communicate the haptic properties of fabrics compared to static stimuli (images), because of the greater availability of information (Bouman, Xiao, Battaglia, & Freeman, 2013; Wijntjes, Xiao, & Volcic, 2019). Xiao et al. (2016) found that when observers can only rely on images to infer the material properties of fabrics, color and folding information interact to enhance the accuracy with which tactile properties are estimated. In the absence of folds, that is, if the fabric is shown flat, chromatic information was found not to be discriminative enough. In perception-based computer graphics, it has been shown that the optical appearance of different fabrics contributes to the realism of the rendering more than their dynamics (Aliaga, O'Sullivan, Gutierrez, & Tamstorf, 2015). The digital rendering of fabrics is gaining importance in the entertainment industry for movies and games (Zhao, Luan, & Bala, 2016) and in online shopping with the option to virtually try on clothes (Pons-Moll, Pujades, Hu, & Black, 2017).

Velvet and satin have different mechanical and optical properties that give rise to their distinctive appearances. The appearance of velvet is due to asperity scattering, where light is scattered by the hairy layer on the surface, leading to a brightening of the contours (Koenderink & Pont, 2003; Pont & Koenderink, 2003). The reflectance properties of satin, which lead to its shiny appearance, depend on its constructional parameters (e.g., the yarn density and the weave pattern) (Akgun, Becerir, & Alpay, 2014). In particular the weave pattern of satin is based on “floating” yarns, yarns that are weaved vertically over a horizontal weft. These floating yarns reflect the light from the fabric creating specular or split-specular reflections causing the shiny appearance (Barati et al., 2015). The specular peaks for satin are located at the regions of highest curvature, and under generic lighting conditions are pointed towards the light source. For velvet, however, the brightest regions are typically placed along its occluding contours, under generic lighting condition (Barati et al., 2015). The position of highlights, being related to the three-dimensional shape of the object, also reveals the folding configuration of the fabric. This folding configuration is informative when estimating the optical and mechanical properties of a piece of fabric when presented with visual information only (Xiao et al., 2016).

Some physical properties of an object or material, such as softness or warmth, are not directly apparent by the optical cues present in the image. To infer these properties, the human visual system can either employ a bottom-up or a top-down approach. The first relies on the profile of image features that triggers material perception. The second approach would first require recognizing the object and the material class it belongs to, and then inferring the material attributes via prior knowledge and learned associations. However, it is not always necessary to identify the object in order to infer the material attributes. Schmidt, Paulun, van Assen, & Fleming (2017; Schmidt, 2019) showed that identifying the material class already provides enough cues to derive material attributes via an “associative approach.” They conducted a rating experiment of several material attributes using unfamiliar shapes rendered with materials with different optical properties (e.g., marble, steel, velvet, etc.). They found that softness estimation relied on recognizing the different materials via the associative approach (e.g., it is velvet, therefore it is soft). These two approaches, that is, bottom-up and top-down typically, but not necessarily exclusively, use local and global visual information, respectively. This then raises the question whether material perception relies on global or local visual information or a combination of both. According to Schwartz and Nishino (2017), material attributes are inherently local, which is why a classifier trained on human similarity judgements could recognize these attributes from small image patches, like image crops. Marlow and Anderson (2013) proposed that human perception of glossiness depends on local image features of the highlights, such as coverage, contrast, and sharpness, but these features are in turn dependent on the global information of the shape and the illumination environment. Balas, Auen, Thrash, and Lammers (2020) proposed that the use of global or large scale visual information for material perception is developed with age, as they found that children's performance in distinguishing between real and fake food was impaired when local information was disrupted but that this impairment was reduced or even absent when global information was disrupted. Schmidt, Fleming, and Valsecchi (2020) showed that local shape features affect the visual perception of softness and weight of unfamiliar, static objects. It is evident from the literature that the understanding of the visual systems’ use of local versus global visual information is still an open problem; therefore in this article we tested and compared material perception providing either global or local information.

Another field in which it is relevant to distinguish and identify different fabrics is art history, because every element within paintings usually carries meaning. For example, in some drawings made around 1490 by a German artist known as the Master of the Coburg Roundels, the lively “fluttering loincloth” of the crucified Christ may signify his imminent resurrection (Lehmann, 2015). Another example is the dress of Eleanor of Toledo, painted by Bronzino in 1546, which symbolized the wealth and power of Florence and the de Medici family in the 16^th^ century (Thomas, 1994). According to Thomas (1994), “in order to understand the origin and purpose of the dress, we must first know the nature of the fabric,” and he wondered whether the fabric was velvet or satin. The original hypothesis that the fabric was brocaded satin was later confirmed when the tombs in de Medici's mausoleum were opened, because the dress was the burial gown of Eleanor of Toledo (Thomas, 1994). However, it should be noted that art historical examples where the depictions of an object or material can actually be compared with the original object or material are for obvious reasons extremely rare.

The first aim of this article was to determine the perceptual material signatures of velvet and satin depicted in 17^th^ century paintings. In Experiment 1, we further explored whether cropping the fabric out of its global form and providing only local information caused a change in perception of its material properties. This indeed happened. In Experiment 2, we investigated whether the observed changes in material perception when judging a cropped image could be related to the choice of the cropped area because of the presence or absence of triggering image cues. Finally, to explore which cues observers relied on to make their judgments, we correlated the perceived material properties in the different crops with image features of the highlights.

## Experiment 1

### Methods

In Experiment 1 six material attributes were rated for a set of paintings of fabrics, depicting either velvet or satin, to measure the extent of association of each attribute with the two types of fabric. The stimuli were presented in two viewing conditions, either with context where the full figure was presented or without context/object shape information, where crops of the fabric were presented. The different viewing conditions were aimed to test whether showing a fabric embedded in a recognizable object, such as a dress or a tablecloth, rather than in an anonymous form without context, would affect the perception of the material attributes.

#### Stimuli

We selected 19 fabrics from 17 high-resolution digital images of 17^th^ century oil paintings. Two paintings depicted both velvet and satin and were therefore used twice. All paintings reproduced within this paper are available under open access at a CC 1.0 or CC BY 4.0 license. The full list of all paintings used within this study, including those reproduced in this paper, can be found in Supplementary Figure S1 in the supplementary materials.

**Figure 2.**
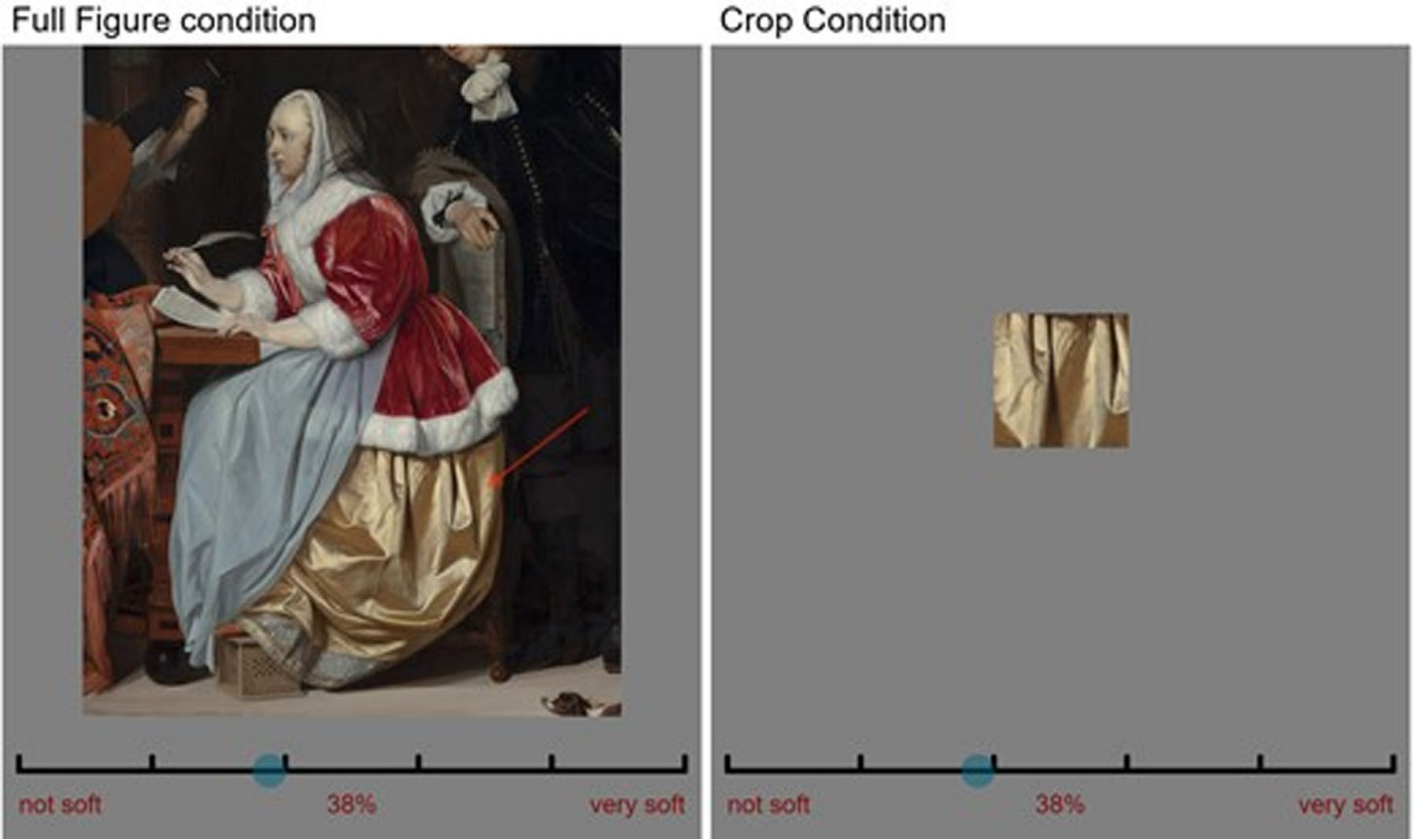
An example of each of the two conditions, within the interface. (Left) The full figure condition, in which the figure or object with the target fabrics is fully visible. On the right, the crop condition, where only a patch from the target fabric is visible, which is intended to deprive the visual system from context and shape information. Note that a participant would see only the left or right screen, never both. Gabriël Metsu, *A Young Woman Composing a Piece of Music*, 1664, Mauritshuis.

The fabrics were categorized as either velvet (n = 8) or satin (n = 11) by the experimenters. The categorization was based on the expertise of all the authors in vision science and optics. We further supported this categorization with art historical sources identifying the fabrics of some of the paintings in our set of stimuli, as either satin or velvet (Gordenker, 1999; Liedtke, 2011; Pottasch, 2020). In one viewing condition, the entire figure or object, including the background, was shown with a red arrow indicating the target fabric to rate (see the left image in Figure 2). In the other viewing condition, each target fabric was cropped to a 600 × 600 pixels patch and presented on the screen at the same visual size as in the full figure condition, against a gray background (see the right image in Figure 2). The cropped areas were chosen to be as informative as possible about the folding shapes. Throughout the rest of the article, we will refer to the two viewing conditions as full figure condition and crop condition, respectively. See Supplementary Figure S1 in the supplementary material for all the stimuli in both viewing conditions.

#### Observers

Each participant rated all the stimuli in one viewing condition and for one material attribute. We collected data from 10 participants for each combination of the two viewing conditions and six attributes, for a total of 120 participants. Data were collected through the Amazon Mechanical Turk (AMT) platform. Although AMT provides some benefits over conventional laboratory settings, it is known to possibly result in noisy data as a result of a small, but considerable portion of participants that appear to perform badly in experiments. On the basis of previous experience with the AMT platform (Van Zuijlen, Pont, & Wijntjes, 2020), we set an exclusion criterion to automatically remove data from participants whose median trial time was below one second (i.e., responding too fast). For each participant removed this way, we collected one more participant until we reached the targeted 10 participants per viewing condition/attribute combination. In total, 48 participants were removed this way, which in hindsight signals that this exclusion criterion might have been too strict. Participants were excluded in this way before any data analysis was performed. All participants were naïve to the purpose of the experiment. They agreed with the informed consent before the experiment. The experiments were conducted in agreement with the Declaration of Helsinki and approved by the Human Research Ethics Committee of the Delft University of Technology.

#### Procedure

Experiment 1 consisted of a between-subjects design, with two viewing conditions and six perceptual attributes, namely roughness, shininess, softness, weight, warmth, and hairiness. Before starting the experiment, participants received written instructions explaining the task. They were informed that they would be shown images of fabrics but not which type of fabric. Before the actual experiment, participants performed 15 practice trials, not only to become familiar with the interface but also to get an idea of the range of stimuli. Participants were randomly assigned to one of the viewing conditions and they were asked to rate one of the attributes. Each attribute was rated using a slider on a continuum ranging from 0 to 100: smooth versus rough, matte versus shiny, hard versus soft, cold versus warm, hairless versus hairy, and light versus heavy. In both viewing conditions, each of the 19 stimuli was rated three times for a total of 57 trials. The trials were randomized across participants.

### Results

#### Consistency between and within observers

**Figure 3.**
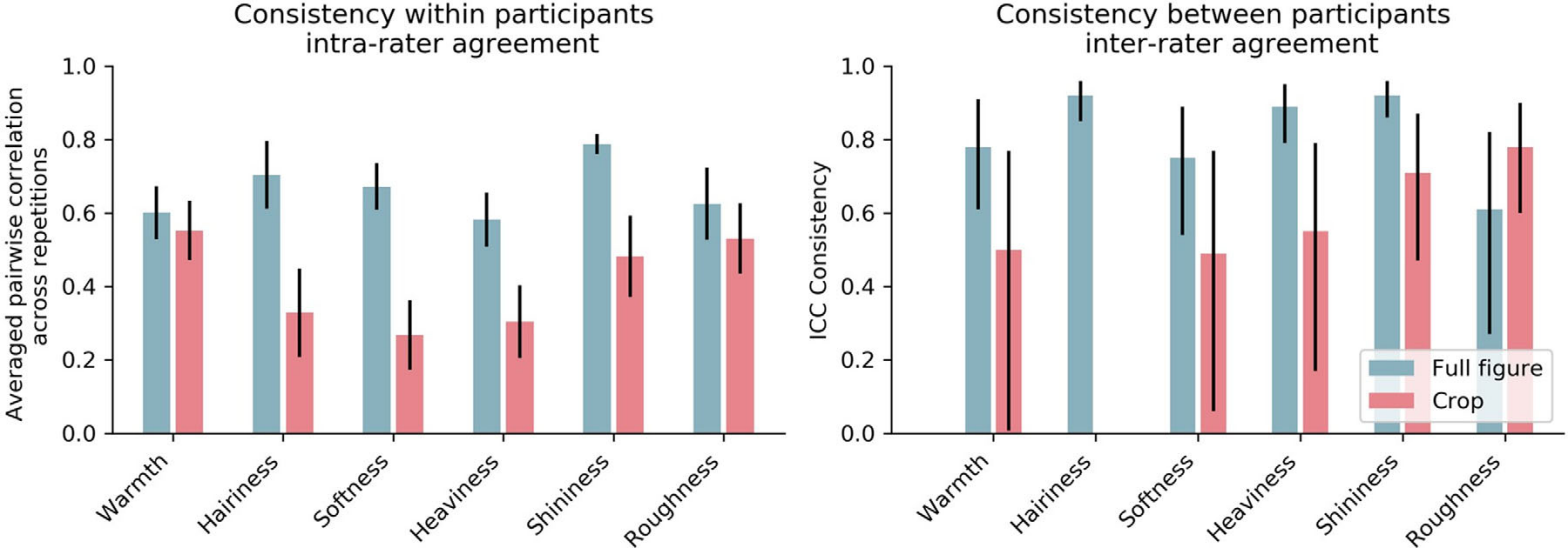
Consistency within and between participants. (Left) The consistency within participants is calculated as the averaged pairwise correlation between each participants repetitions of the stimuli, and the error bars indicate the standard error. Right) The consistency between participants was calculated using intraclass correlations, and the error bars indicate the 95% confidence interval. The full report of the ICC analysis can be found in Table S1. Note that non-significant ICC are not visualized (i.e., hairiness in the crop condition).

In Experiment 1 each attribute was rated three times. The consistency within observers is visualized in Figure 3 (left) and was calculated as the average pairwise (Pearson) correlation between the ratings over the three repetitions per observer, again averaged across observers. Next, we took the median across the three repetitions to smooth out the effects of potential outliers. Then, we normalized the data for each participant between 0 and 1 to rule out possible effects of unequal interval judgments. We used this median, normalized data for the remainder of the result section. For the consistency between participants, we calculated the intraclass correlation coefficient (ICC) using an average rating, consistency, two-way random effects model for each attribute and each condition (McGraw & Wong, 1996; Koo & Li, 2016). The ICC values and the 95% confidence intervals have been visualized in Figure 3 (right). A full report of the ICC statistics can be found in Supplementary Table S1. In Figure 3 there is a clear trend of higher interrater and intrarater agreement in the full figure condition compared to the crop condition, with the exception of roughness in the interrater agreement (Figure 3, right). For the ratings of roughness, some participants in the crop condition may have attended to the visible roughness of the brushstrokes instead of judging the fabric. Furthermore, the ICC calculations show that the consistency between participants is significantly different from zero, thus above chance, for all attributes and in both viewing conditions, with the only exception of hairiness in the crop condition. However, the intrarater agreement on hairiness was high and significant in both viewing conditions.

#### Material signatures

**Figure 4.**
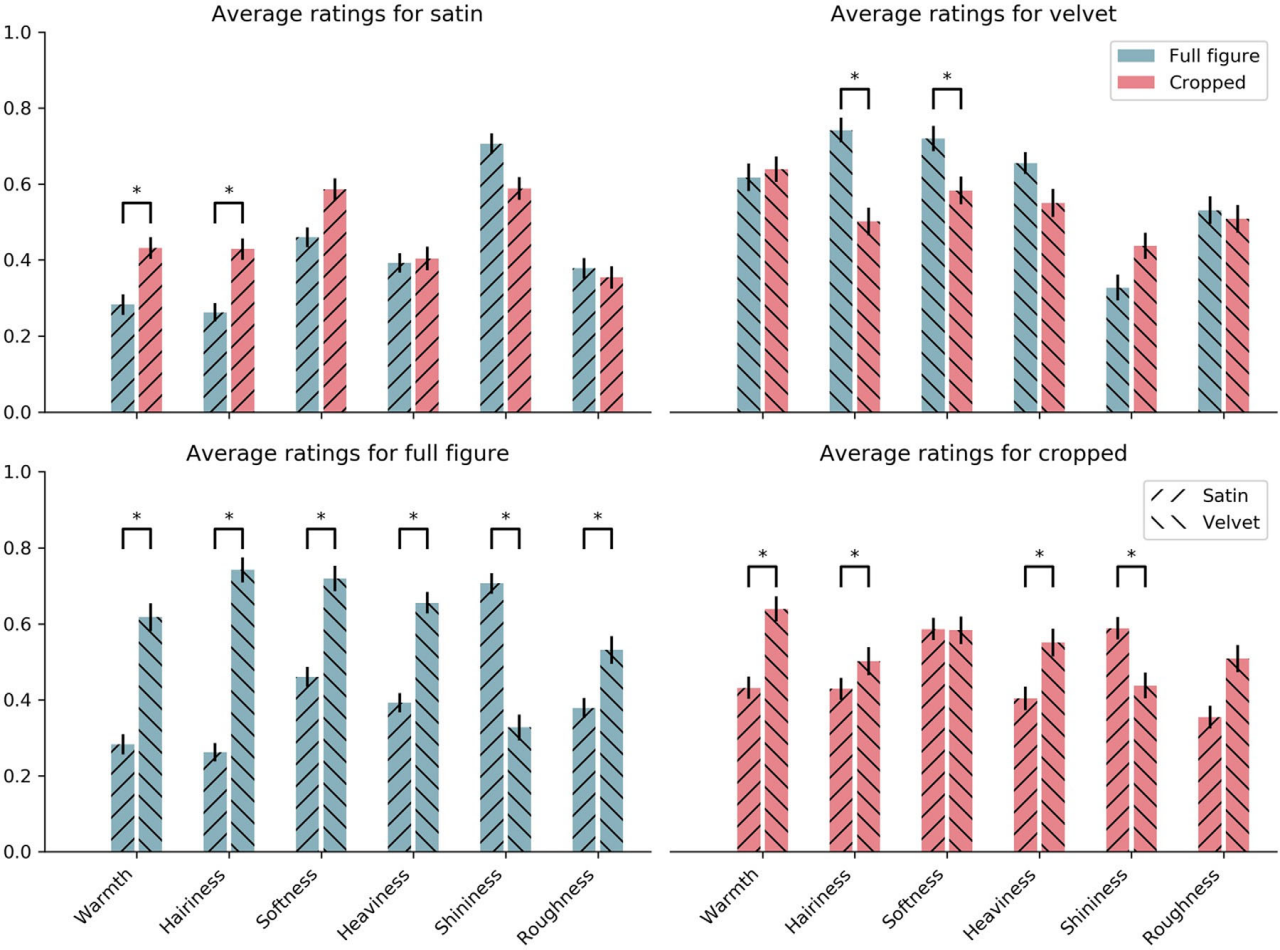
The perceptual judgments of satin and velvet, for both conditions. In the top plots, the data are split by viewing condition, whereas in the bottom plots data are divided by material. For each participant, we took the median rating across the stimuli repetitions, and then averaged across these values. Significance between condition (top) and material (bottom) is indicated at *p* < 0.05, Bonferroni corrected. Note that besides the significance, the top and bottom display the same data, only differently presented to make interpretations across conditions easier, and to avoid visual clutter of displaying all significant differences within a single plot.

We ran a two-way multivariate analysis of variance (MANOVA) to examine the effect of the viewing condition and the fabrics’ material on the perception of the material attributes. We found a main effect for both viewing condition (i.e., full figure vs. crop) at *F*(6, 29) = 2.78, *p* < 0.05, and material (i.e., velvet vs. satin) at *F*(6, 29) = 23, *p* < 0.001. We also found an interaction effect between the two factors at *F*(6,29) = 6.56, *p* < 0.001. In Figure 4, we visualized the average judgments of the material attributes, split by viewing condition (top) and material (bottom) and indicate significant differences (Bonferroni corrected) between the conditions. The perception of warmth and hairiness of satin, and hairiness and softness of velvet changed significantly between the two viewing conditions. For the full figure condition, velvet was judged to be significantly warmer, hairier, softer, heavier, and rougher, whereas satin was perceived to be shinier. For the crop condition, velvet was significantly warmer, hairier, and heavier, whereas satin was rated significantly shinier. There were no significant differences between satin and velvet, for the attributes of softness and roughness, in the crop condition.

**Figure 5.**
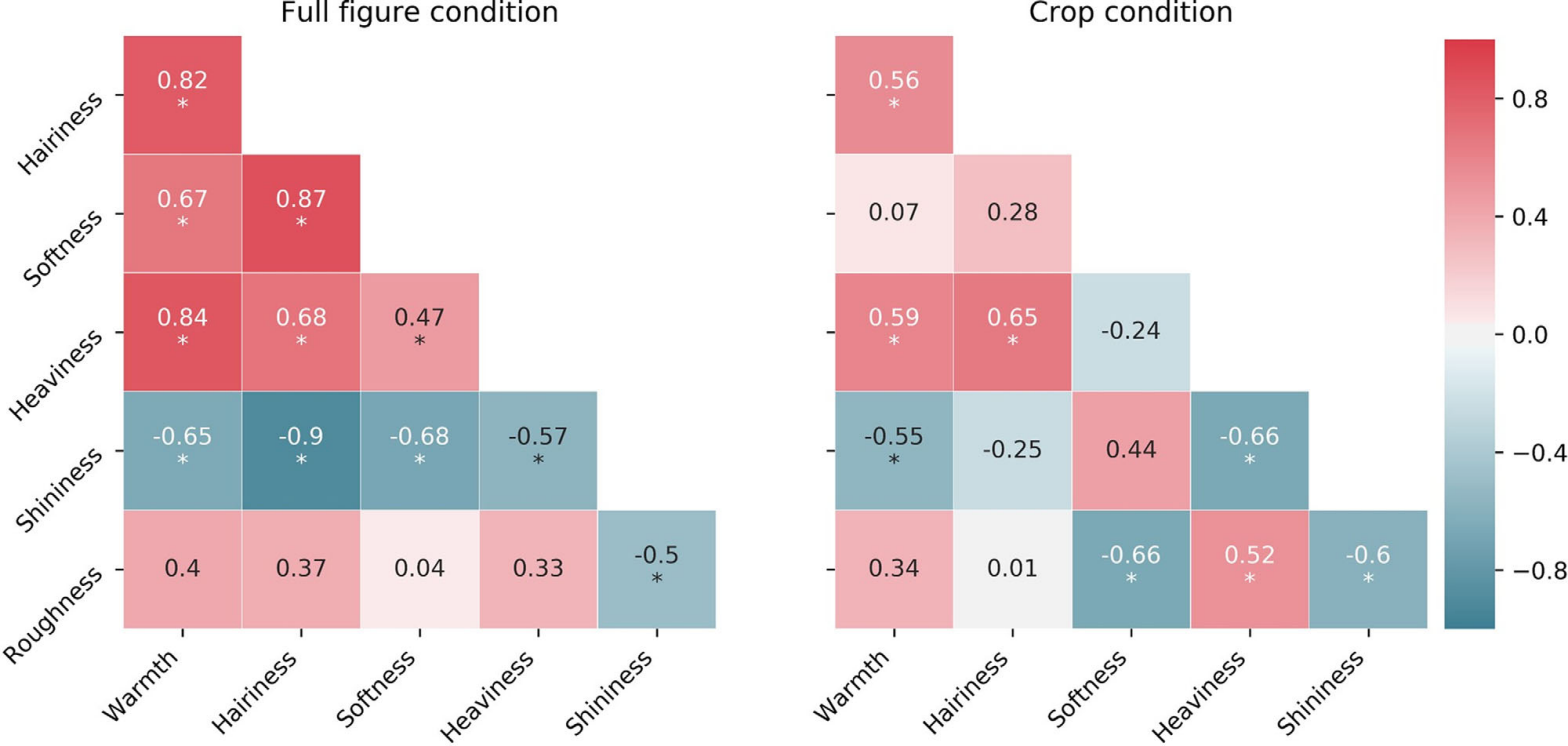
Correlation matrices of the attributes for both conditions. Color indicates the magnitude of the correlation coefficient. Asterisk (*) indicates a significant effect at *p* < 0.05.

To check whether the material attributes were independent of each other or belonged to an underlying subset of dimensions, we computed a correlation matrix for both viewing conditions, visualized in Figure 5. The correlation coefficients are reported in the cells of the matrices. Significant correlations at *p* < 0.05 are marked with an asterisk (*).

In the full figure condition, shininess was the only attribute that showed a negative significant correlation with each other attribute. All other attributes showed mutual positive, significant correlations except for roughness, which only correlated (negatively) with shininess.

In the crop condition, fewer correlations were found across all attributes. Roughness was again negatively and significantly correlated with shininess, as well as with softness and positively correlated with heaviness. Shininess was no longer correlated with hairiness, nor softness. Overall, this shows that the material attributes are not completely independent of each other, which implies they might be captured by a smaller set of dimensions.

#### Principal component analysis and Procrustes analysis

**Figure 6.**
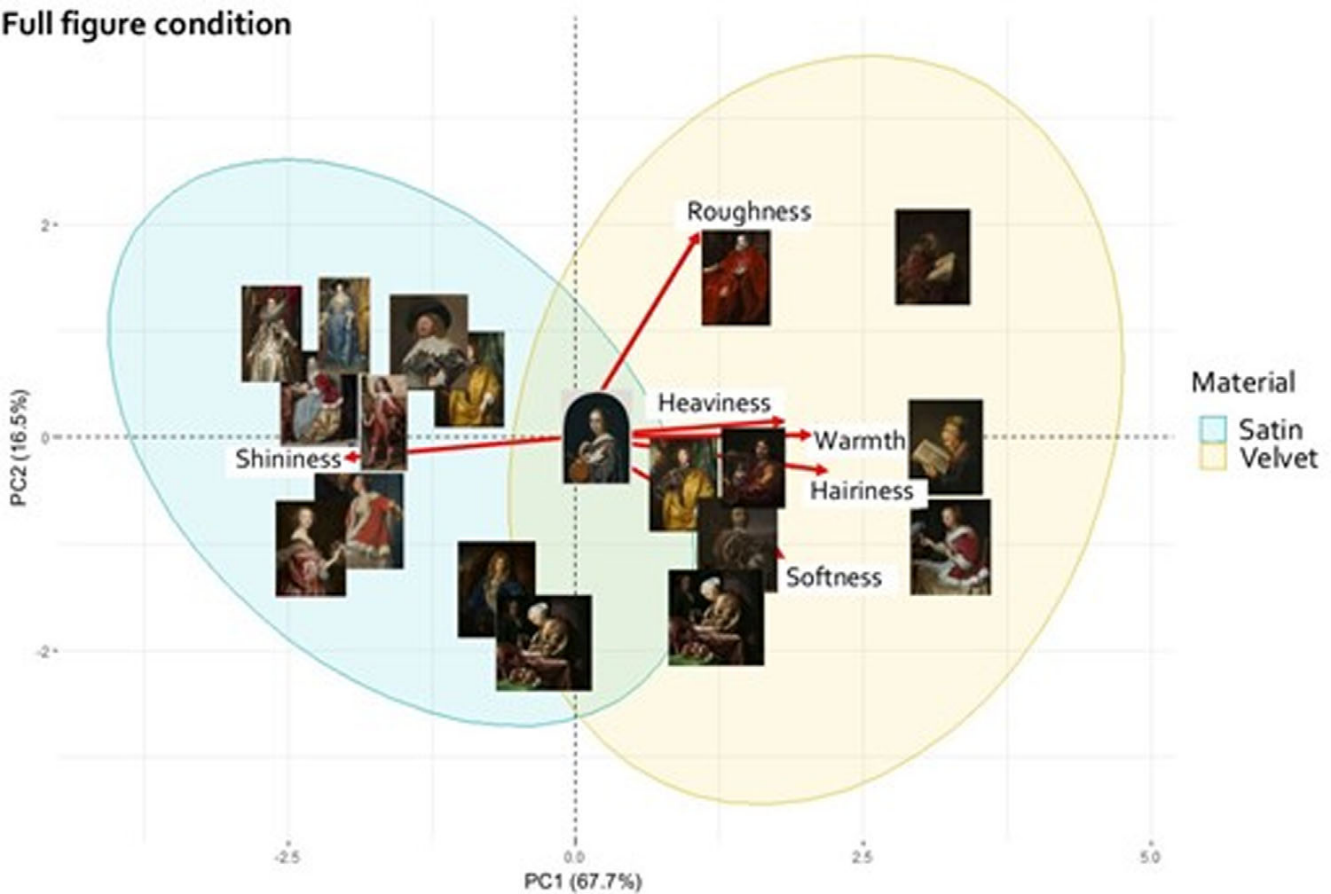
PCA biplot for the full figure condition. The materials are clustered within 95% confidence ellipses. Attributions of all stimuli can be found in Supplementary Figure S1 in the supplementary materials.

**Figure 7.**
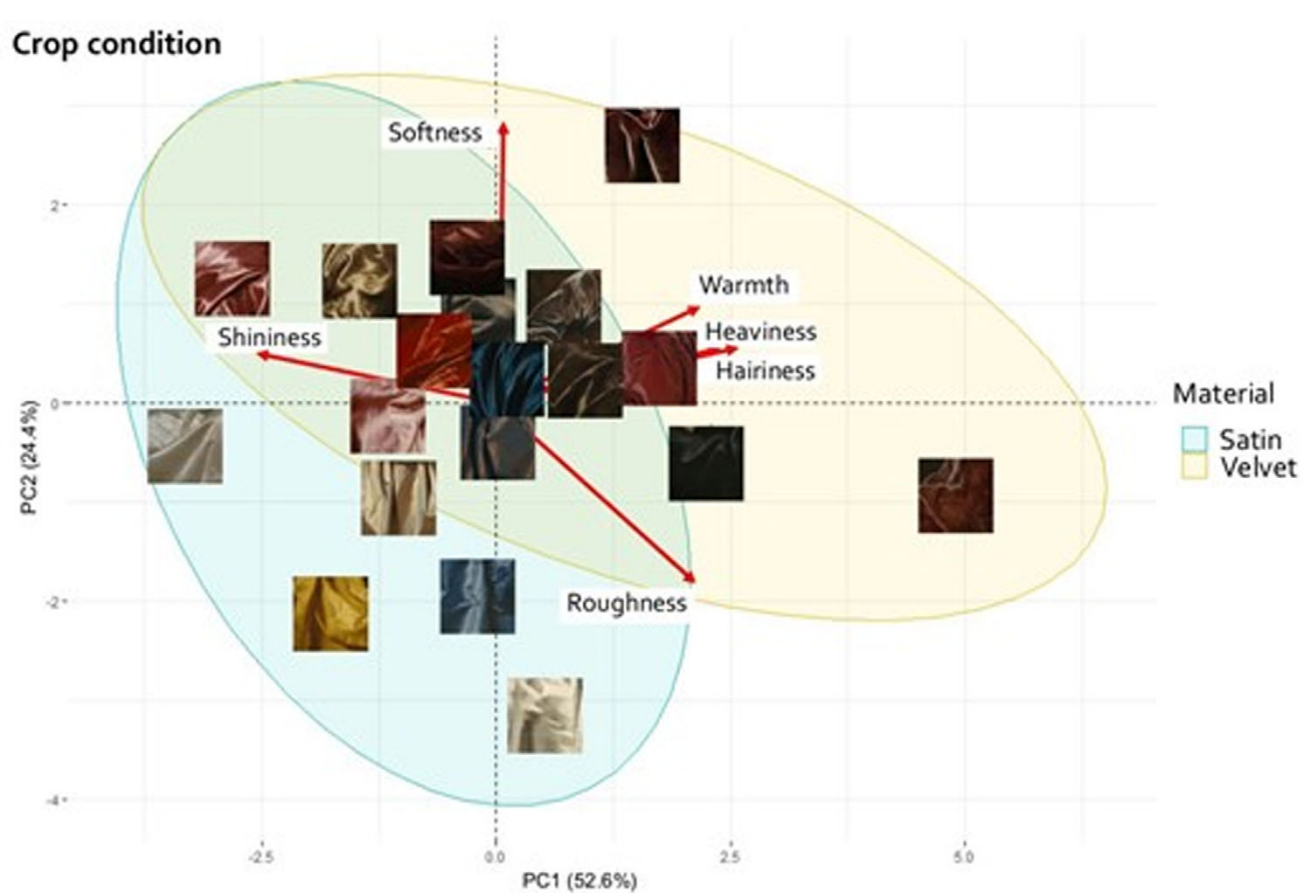
PCA biplot for the crop condition. The materials are clustered within 95% confidence ellipses. Attributions of all stimuli can be found in Supplementary Figure S1 in the supplementary materials.

To visualize whether the two materials, velvet and satin, were perceived as having different material properties, we ran a principal component analysis (PCA) for both viewing conditions. Figures 6 and 7 show the PCA biplots of the full figure and the crop conditions, respectively. These biplots indicate how the stimuli are related to the attributes. The stimuli were clustered using 95% confidence covariance ellipses, according to the depicted material, satin (light blue ellipse), or velvet (yellow ellipse).

To further compare the effect of cropping on the material properties perception, we performed Procrustes analysis. The PCA of the crop condition shown in Figure 7 was matched to the PCA of the full figure condition (Figure 6).

In the full figure condition, the first two principal components account for 84.2% of the variance. The factor loadings listed in Table 1, show that the first principal component is positively loaded by a cluster of attributes including hairiness, warmth, and heaviness. In the negative direction, shininess loads on the first component. The second principal component is mostly loaded by roughness.

**Table 1.** The factor loadings for the first two principle components of two PCAs, one for each condition.

	PC1 full figure	PC2 full figure	PC1 crop	PC2 crop
Warmth	0.45	0.01	0.41	0.27
Hairiness	0.48	−0.14	0.49	0.16
Softness	0.39	−0.50	0.015	0.78
Heaviness	0.40	0.06	0.45	0.15
Shininess	−0.44	−0.1	−0.48	0.14
Roughness	0.24	0.85	0.40	−0.51

In the crop condition, the first two principal components explain 77% of the variance. The first component is mostly loaded in the positive direction by hairiness, heaviness, and warmth and by shininess in the negative direction. The second component is mostly loaded positively by softness and negatively by roughness.

**Figure 8.**
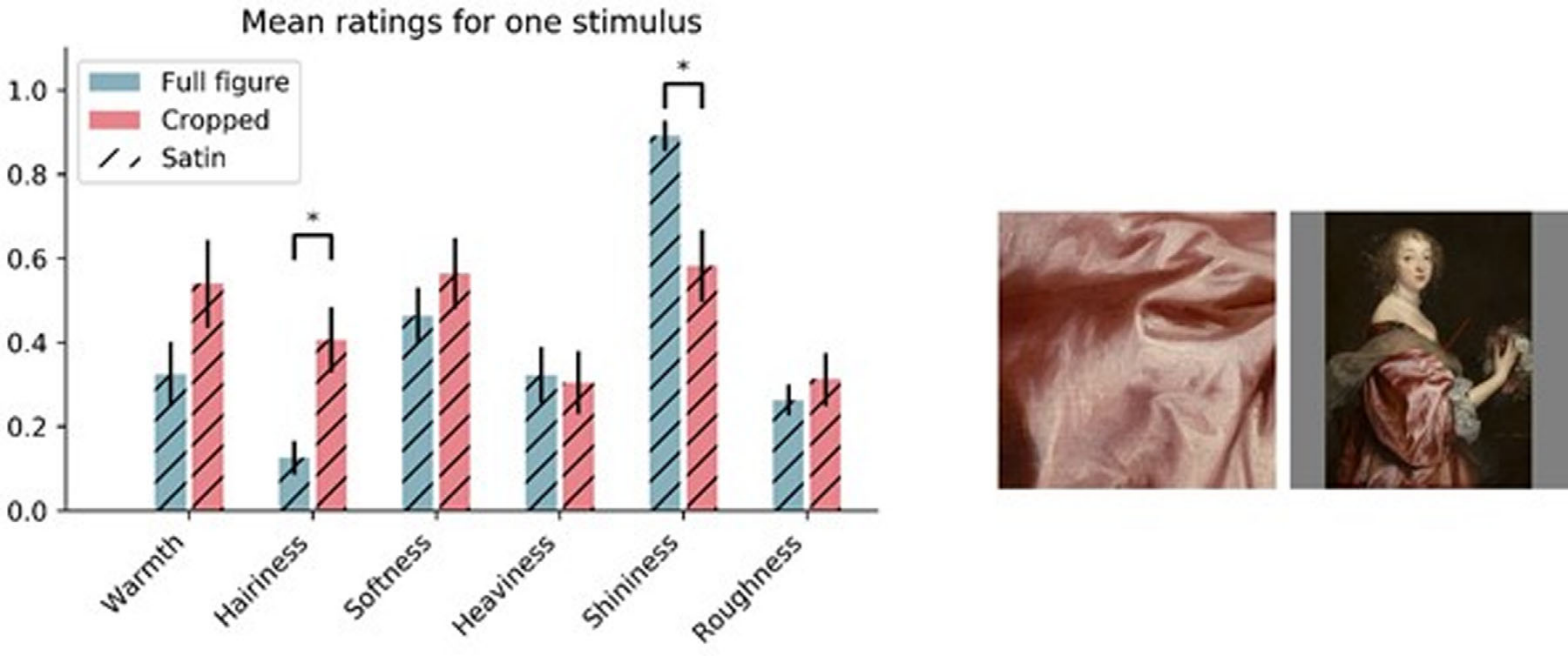
Left: The mean ratings on the y-axis of the attributes for one specific stimulus. An asterisk (*) indicates a significant difference at *p* < 0.05. Right: the crop and full figure stimuli represented in the left bar chart. Error bars indicate the standard error. Anthony van Dyck, *Catherine Howard, Lady d'Aubigny*, 1638, National Gallery of Art.

A permutational test to check the significance of the Procrustes result (*r* = 0.72, *p* < 0.001), indicated that the overall distribution of the stimuli was similar between the PCA of the full figure condition and of the crop condition. However, the distribution of the stimuli in the PCA biplot (Figure 7) shows much more overlap of the velvet and satin clusters, compared to the PCA of the full figure condition (Figure 6). In addition, some stimuli clearly changed location between the two PCA spaces, indicating that their perception differed in the two viewing conditions. One example is shown in Figure 8. The mean ratings of all the attributes for this fabric, averaged over the median rating of each participant, are shown in Figure 8 for the two viewing conditions. The asterisk indicates that hairiness and shininess were perceived to be significantly different at *p* < 0.05 between the two viewing conditions.

### Intermediate conclusions and discussion

We conclude that, within the attributes that we tested, the material signature of depicted velvet included warmth, heaviness, hairiness, and softness, and the signature of depicted satin included shininess. We further conclude that depriving the visual system of context and shape information significantly changed the perception of fabrics depicted in 17^th^ century paintings. Specifically, when depriving the visual system of shape and object information, the perception of material attributes can drastically change, as exemplified in Figure 8. Moreover, the percepts became less consistent and more subjective as observed from the decrease in both interrater and intrarater agreement. Furthermore, differences between materials expressed as the distributions of perceived material attributes became less distinct.

The cropped areas shown in the crop condition were chosen according to the amount of folding, in an attempt to maximize the amount of visual information. Considering that, we wondered to what extent the differences in perception found between the crop and full figure conditions were affected by this choice. One could argue that the depicted dress or robe from which crops are taken presents a certain shininess, roughness, and more, and thus different crops from it would present these properties quite consistently without qualitative changes in perception between crops. However, on the other hand, local variations in shape (drapery) and effective lighting can cause major appearance variations, thereby causing differences in perception between the full figure condition, where participants could attend to all image features anywhere on the clothing and the selected crop condition. For instance, a crop that coincidentally captures many highlights might be perceived to be shinier relative to a crop with few or no highlights, and, vice versa, it might also be possible that key image features were absent in our crops. We follow up on this question in Experiment 2 where we tested if the perception of crops changed depends on the choice of cropped area.

## Experiment 2

### Methods

In Experiment 2, we investigated the extent to which perception of material attributes varies depending on the content of the crop and the presence or absence of local image features. We tested this with two material attributes that we also used in the previous experiment. The experiment consisted of a rating task of the two material attributes, followed by image analysis of the crops to extract highlights’ features that could relate to the variations in perception between crops of the same fabric.

#### Stimuli

**Figure 9.**
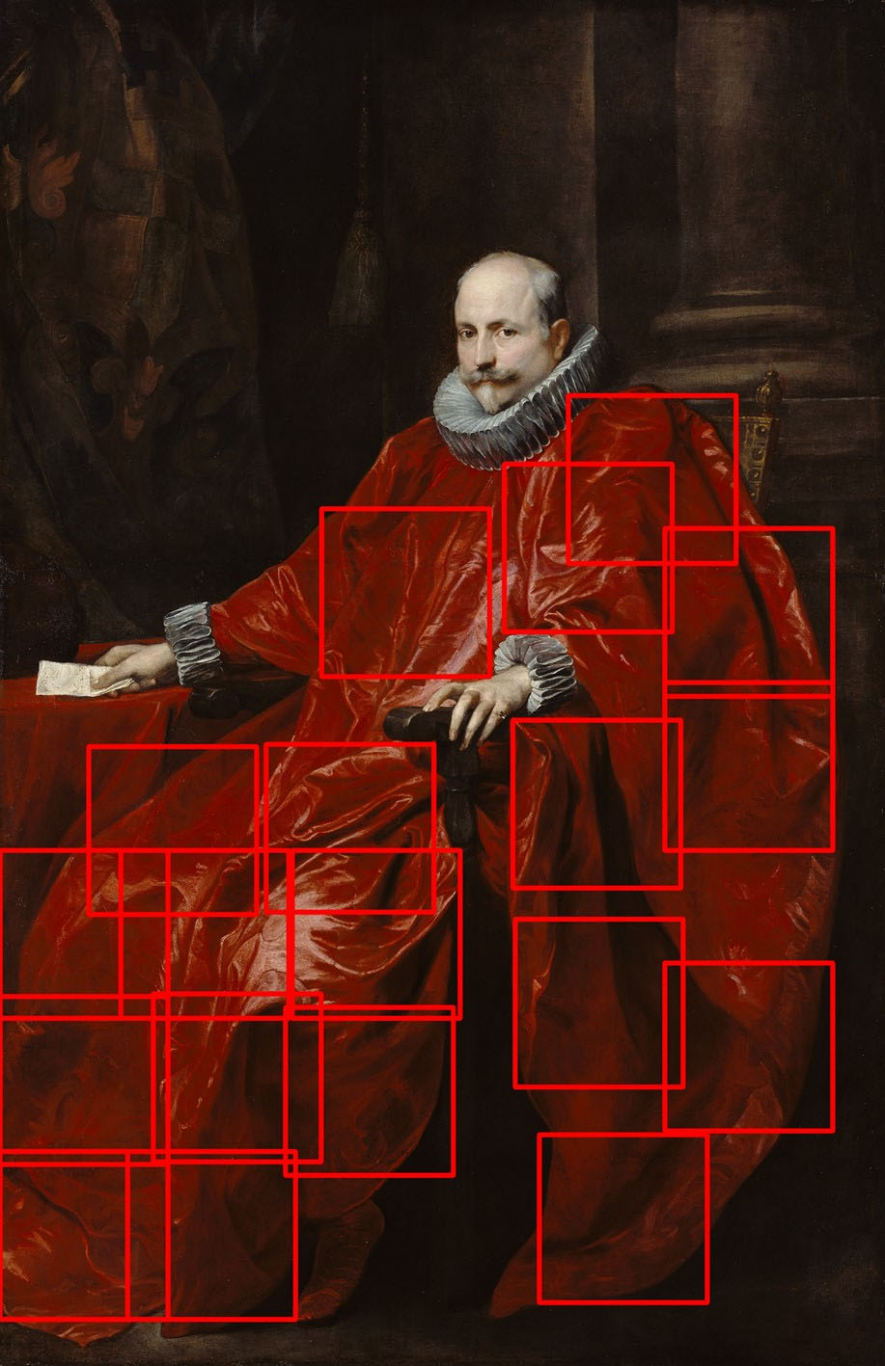
The original full figure stimuli, with red boxes that indicate the crops made for this stimulus. Each of the 19 stimuli from Experiment 1 was subdivided into a set of crops as shown here. These sets of crops were used as stimuli in experiment two. Each crop within a set was the same size. Anthony van Dyck, *Portrait of Agostino Pallavicini*, 1621, J. Paul Getty Museum.

We used the 19 fabric stimuli from the full figure condition in Experiment 1 to make the stimuli in Experiment 2. From each image, we extracted a set of nine to 21 equally sized crops, which covered the whole fabric (see Figure 9 for an example). Thus we made 19 sets of crops (velvet n = 8 and satin n = 11). To keep the visual size of the folds in the crops as consistent as possible across different sets, the images were cropped with a constant ratio between the width of the whole fabric in the original image and the width of the crops. Images of all the crops can be found in the supplementary materials Supplementary Figure S2.

#### Observers

Identical to Experiment 1, data were collected on the AMT platform. Each of the 19 sets of crops was judged by a group of 5 participants for either shininess or softness. That is, participants would rate one set of crops for one material attribute. A total of 190 AMT users participated in the second experiment. All participants were naïve to the purpose of the experiment, and none had participated in the first experiment. Each participant agreed with the informed consent before performing the experiment. The experiments were conducted in agreement with the Declaration of Helsinki and approved by the Human Research Ethics Committee of the Delft University of Technology.

#### Material attributes

We used two material attributes in this experiment, both of which were also measured in Experiment 1. The first attribute was shininess, and the second was softness. Softness was found to be not correlated (see Figure 5) with shininess, and it can be seen to be nearly perpendicular to shininess in the crop condition PCA (Figure 7). We interpreted this to mean that the majority of variability captured by softness is not explained by shininess, and vice versa, and that these two represented two main underlying dimensions of a perceptual material attribute space. Roughness was found to not be significantly different between velvet and satin and thus is unlikely to represent an underlying feature in this material space. The three remaining attributes used in Experiment 1 (warmth, hairiness, and heaviness) all intercorrelate and likely compose one underlying dimension. Therefore with choosing shininess and softness we hope to capture the majority of the variation and underlying dimensions of the material feature space for fabrics with the least amount of attributes.

#### Procedure rating experiment

In Experiment 2, participants were asked to rate one material attribute for each crop in one set of crops, taken from one of the 19 fabrics used in Experiment 1. After having read the instructions and having agreed to the informed consent, participants were asked to perform a size calibration, by adjusting a digital image of a credit card until it matches a physical payment card in the possession of the participants. Because all payment cards adhere to the standard set size forth by the International Organization for Standardization's 7810 ID-1 format (ISO/IEC 7810-ID-1), this allows us to rescale all images, so that each stimulus was presented at the same size, across different display settings for different participants. After the size calibration, participants performed a 10-second free-viewing task of the crops to get an idea of the range of the stimuli. Next, participants performed five practice trials followed by the actual experiment. For each trial, participant were tasked with rating shininess or softness with a slider on a continuum ranging from 0 to 100, corresponding to matte to shiny and hard to soft, as in Experiment 1. Each crop was rated three times, for a total number of trials ranging from 27 to 63 depending on the number of crops. The trials were randomized across participants.

#### Procedure image analysis of highlights

One way painters distinguished the depiction of velvet from satin is through the rendering of the key image features of their reflectance properties (Gombrich, 1976). We hypothesized that, when judging the material properties of such depicted fabrics, humans attend to similar image features as perceptual cues.

Via photometric measurements of fabric samples, Barati et al. (2015) assigned satin to a reflectance category combining specular and split-specular scattering, and velvet to the category of asperity scattering materials. From a perceptual rating experiment, Barati et al. (2015) also found that the samples belonging to the asperity scattering category were perceived to be the softest, whereas the samples in the specular and split-specular scattering class were perceived to be the shiniest and the least soft. These findings support our hypothesis that softness and shininess are key attributes of velvet and satin, respectively.

The different scattering behaviors of velvet and satin result in distinctive optical cues. Previous studies have shown that image features of the highlights, such as coverage, contrast and sharpness, can influence the perception of glossiness (Marlow, Kim, and Anderson 2012; Marlow & Anderson, 2013; Qi, Chantler, Siebert, & Dong, 2014; Di Cicco, Wijntjes & Pont, 2019; Schmid, Barla & Doerschner, 2020). To test whether the perception of shininess and softness depended on the choice of the cropped area, and therefore on the image features of the highlights present in the crop, we computed the mean luminance of the crops, the relative coverage of the highlights and the mean contrast of the highlights. We did not measure sharpness because that was assumed to be relatively consistent between crops of the same painting.

**Figure 10.**
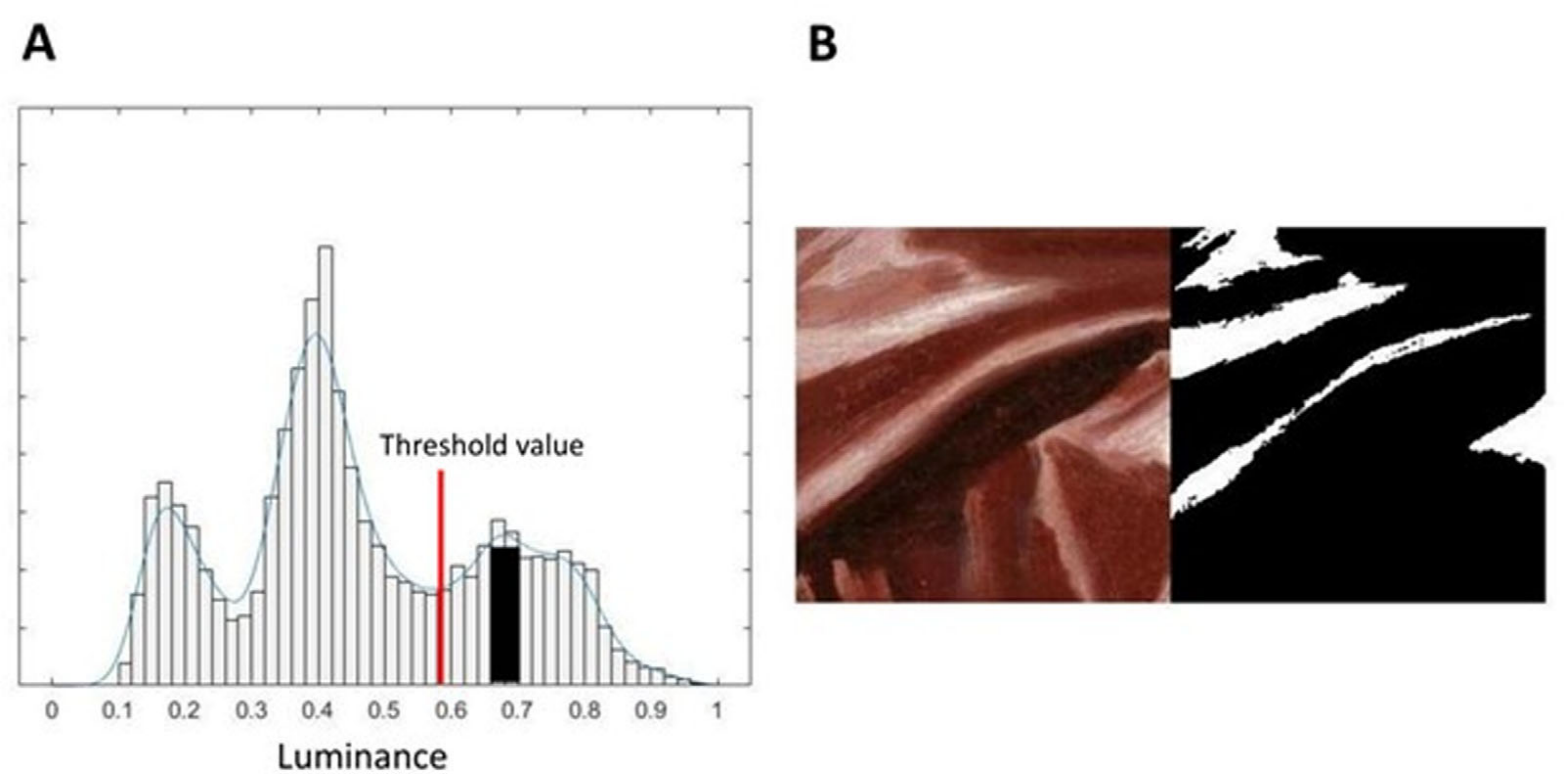
(A) Luminance distribution and the highlight mode used as threshold value (black bar) to create the binarized image. (B) The original stimulus, as presented to the participants in the rating experiment, and its binarized version.

The calculations of the highlight features, that is, coverage and contrast, were done using binary images of the crops. The threshold values to binarize the images and isolate the highlights for the computations, were manually derived from the luminance histogram of each crop. Figure 10 A shows the luminance histogram of the crop shown in Figure 10 B. The highlight mode, one of the three general modes for a histogram-based measure of the surface structure proposed by Pont (2009), is indicated by a black bar (note that here the width and height of the bar have no other meaning beside providing a clear visual indication of the threshold value used to binarize the image, whereas in Pont (2009) these parameters were related to the width and the height of the mode). To binarize the images, we manually selected the threshold at the minimum value of the highlight mode (indicated by the red line in Figure 10A). The manual selection was done for every crop. Figure 10 B shows the original crop and its binary image.

The contrast was calculated as Michelson contrast, using the ninety-fifth and the fifth percentiles of the luminance values instead of the absolute maximum and minimum for robustness; the percentage of coverage was calculated as the ratio of the areas covered by white and by black pixels in the binarized image. All image analyses were done in Matlab 2018a (The MathWorks Inc., Natick, MA, USA).

Note that the measures of the highlights’ features reported here should be considered only rough approximations because of the complexity of automatically and accurately segmenting the image regions that correspond to the highlights, especially in the case of paintings for which the ground truth is not known. Designing a robust algorithm to measure the image features of highlights that is generalizable to natural images such as photographs or paintings, is still an unsolved problem in the literature, due to the difficulty of defining and identifying what the visual system considers to be a highlight. In a previous study (Di Cicco, Wijntjes & Pont, 2019), we addressed this issue by combining manual annotation of the highlights and self-developed algorithms for the semiautomatic computation of highlights’ features directly from images of paintings. However, the paintings analyzed in that study were exclusively depicting grapes, meaning that each object showed a single, mostly round, specular reflection. This simplified the annotation and computation, and made the method more difficult to apply to paintings of fabrics with multiple reflections of various shapes. Marlow, Kim and Anderson (2012) approached the problem by using psychophysical measurements of contrast, coverage and sharpness of highlights. They further compared the human judgements of the highlights’ features with measures obtained via direct image computation, finding high correlations between the two types of measurements. Qi et al. (2014) used a pixel-wise computation of the highlights’ features based on luminance threshold for stimuli rendered with the same reflectance and illumination parameters. Recently, Schmid, Barla and Doerschner (2020) developed a series of image-based calculations of the highlights’ features that could be applied to stimuli with different shapes, but only with rendered images for which the diffuse and specular components can be defined.

### Results

#### Consistency between and within observers

**Figure 11.**
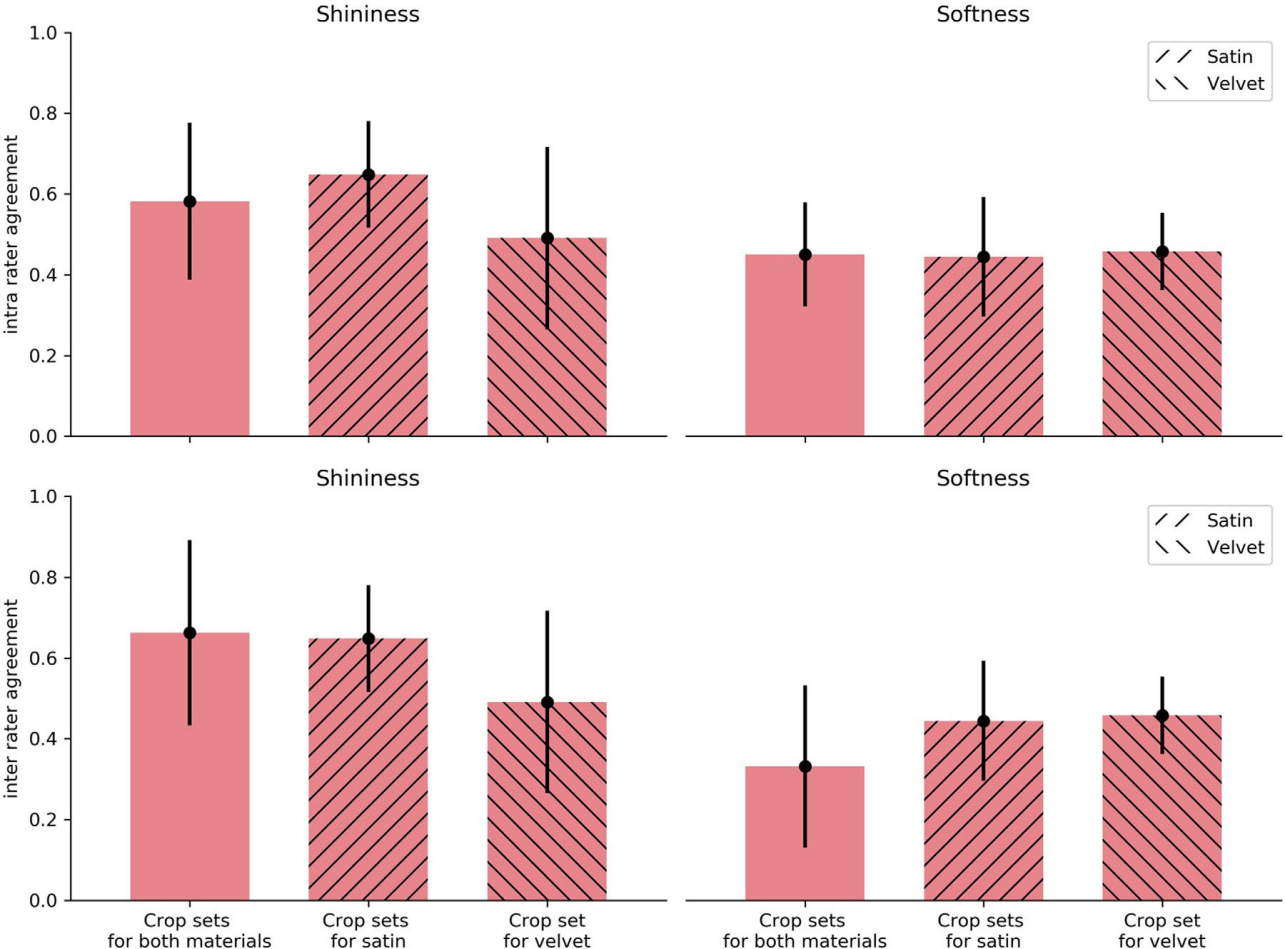
The intrarater and interrater agreement. The top contains the intrarater agreement (consistency within observers) with shininess on the left and softness on the right. The same ordering is applied at the bottom for the interrater agreement (agreement between observers). The error bar indicates the standard deviation.

The intrarater and interrater agreement Figure 11 were calculated for each of the 19 sets of crops for both material attributes. Because shininess and softness were rated three times per crop, before the data analysis we took the median over the three repetitions of the ratings to smooth out the effects of potential outliers. Then, the data were normalized to rule out possible effects of unequal interval judgments. Consistency within observers was calculated as the average correlation between the ratings over the three repetitions for each observer. The consistency between participants was calculated as the mean correlation between all participants.

First, we report the consistency within and between participants split on material attribute. The agreement both within and between participants varied greatly. This indicates that some sets of crops triggered a clear and consistent perception, whereas other sets were perceptually ambiguous.

#### ANOVA

The median ratings of shininess and softness were averaged over all participants for each set of crops, to calculate a one-way analysis of variance (ANOVA) to measure the effect of varying the cropped area. A significant effect for a set of crops indicates that the perceptual ratings differed between crops taken from a single fabric. Significant differences were evaluated at *p* = 0.001 after Bonferroni correction. The results of each individual ANOVA are reported Table 2. Overall, the crops of 15 crop sets were significantly different for shininess, 10 of which depicted satin, and five depicted velvet. Softness was significantly different for only three sets of the crops, two of which depicted satin and the remaining one velvet.

**Table 2.** Results of One-Way ANOVAs of Experiment 2.

Stimuli	Shininess			Softness		
# material		*F*-value	*P* value		*F*-value	*P* value
1 S	F(10, 55)	1.8	>.05	F(10, 55)	0.5	>.05
2 S	F(16, 85)	3.8	<.001^*^	F(16, 136)	1.1	>.05
3 S	F(12, 52)	8.1	<.001^*^	F(12, 91)	3.2	<.01
4 S	F(14, 60)	10.1	<.001^*^	F(14, 45)	0.6	>.05
5 S	F(16, 136)	23.1	<.001^*^	F(16, 85)	6.7	<.001^*^
6 S	F(19, 60)	3.7	<.001^*^	F(19, 60)	9.9	<.001^*^
7 S	F(11, 48)	8.3	<.001^*^	F(11, 84)	5.7	<.01
8 S	F(20, 84)	15.8	<.001^*^	F(20, 126)	1.3	>.05
9 S	F(12, 91)	32.0	<.001^*^	F(12, 65)	0.7	>.05
10 S	F(11, 96)	6.2	<.001^*^	F(11, 84)	1.2	>.05
11 S	F(16, 119)	24.5	<.001^*^	F(16, 136)	1.7	>.05
12 V	F(18, 76)	13.1	<.001^*^	F(18, 133)	0.9	>.05
13 V	F(10, 77)	13.3	<.001^*^	F(10, 66)	2.7	<.01
14 V	F(14, 90)	10.7	<.001^*^	F(14, 75)	0.5	>.05
15 V	F(8, 45)	16.2	<.001^*^	F(8, 27)	0.2	>.05
16 V	F(11, 84)	3.4	<.001^*^	F(11, 72)	1.8	>.05
17 V	F(12, 52)	1.5	>.05	F(12, 52)	5.8	<.001^*^
18 V	F(12, 52)	1.2	>.05	F(12, 117)	0.7	>.05
19 V	F(12, 52)	0.9	>.05	F(12, 52)	0.6	>.05

The numbering of the stimuli corresponds to that of Supplementary Figure S2 reported in italic in the supplementary materials. As can be seen, a significant effect (and thus a varying precept across the same fabric) was found more often for shininess than softness. Stimuli material identity is marked by an S for satin and a V for velvet.

* The ANOVAs significant after Bonferroni correction.

The results from the ANOVAs showed that crops were perceived to vary significantly in shininess within most of the crop sets. We hypothesized that the observed variation in shininess perception can be related to the image features of the highlights available in the different crops.

#### Correlation with highlights’ features

We performed correlation analysis to evaluate the relationships between the mean ratings of shininess and softness of the crops and the features calculated from the images, namely the mean luminance of the crops, and the coverage and contrast of the highlights.

We only performed the correlations for the sets of crops in which we found significant differences with the one-way ANOVA, that is, 15 sets for shininess and three for softness.

**Figure 12.**
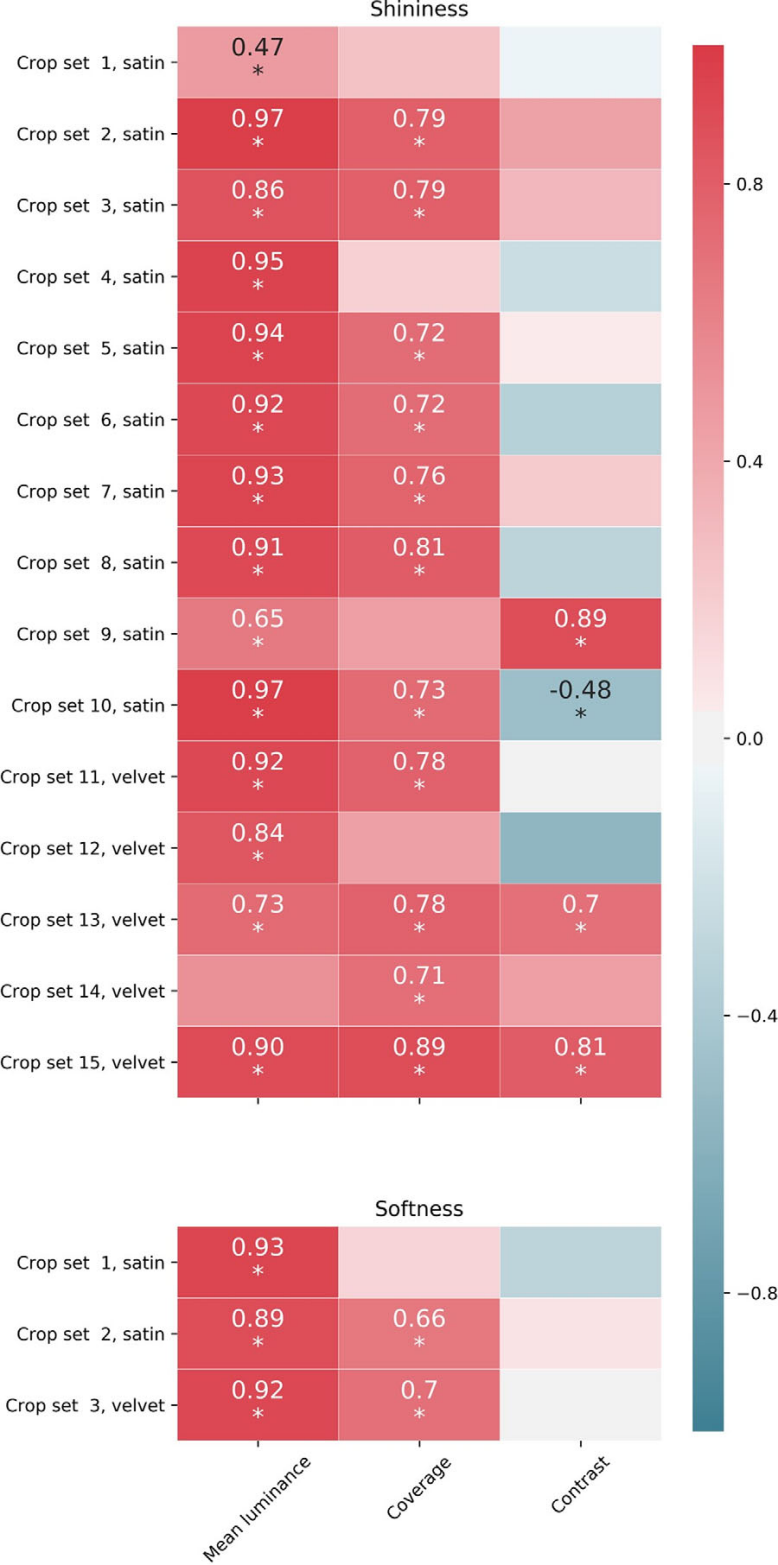
Correlation coefficients of shininess (top) and softness (bottom) with the image features highlights’ contrast, highlights’ coverage, and mean luminance of the crops. The values reported are significant at *p* < 0.05.

In Figure 12 we reported the correlation coefficients of the image features with shininess (top) and softness (bottom). Only the values significant at *p* < 0.05 were reported. The stimuli corresponding to the crop sets are reported in Supplementary Figure S2 in the supplementary material. Note that the crop sets 1 to 3 for softness do not correspond to the crop sets 1 to 3 for shininess.

The top of Figure 12 shows that for 14 of 15 significantly different sets, shininess was positively and significantly correlated with the mean luminance of the crops. For 11 crop sets, shininess was also positively and significantly correlated with the coverage of the highlights. Three of the sets showed a significant positive correlation with the contrast of the highlights, whereas for one set the correlation with contrast was negative and significant.

The three sets with crops significantly different in softness reported in Figure 12, were all positively and significantly correlated with the mean luminance. Two of them were also significantly and positively correlated to the coverage of the highlights. None of them was related to the contrast of the highlights.

## General discussion

In Experiment 1, we aimed to determine which material attributes belong to the signatures of velvet and satin depicted in 17^th^ century paintings. We further tested if removing shape and context information by only presenting crops of the fabric, caused a change in perception. We found that velvet and satin were judged to have different material attributes, as indicated by the two-way MANOVA (Figure 4) and the PCAs (Figures 6 and 7), and that the commonalities in the judgments were based on robust material signatures that are specific for velvet and satin. In the full figure condition, velvet was judged to be warmer, hairier, softer, heavier, and rougher, while satin was perceived to be shinier. In the PCAs for both conditions, shininess appears to be directed towards the satin cluster while the remaining attributes point more towards the velvet cluster. When we look at the velvet and satin clusters in the PCA for the full figure condition (Figure 6) we also see that the materials are separated. In the crop condition (Figure 7), this separation became less, implying that the distinction between satin and velvet decreases in the crop condition relative to the full figure condition. This is also shown in our finding that all material attributes were significantly different between satin and velvet in the full figure condition, but only part of them in the crop condition. This leads to the following result: satin and velvet depicted in 17^th^ century paintings are perceptually distinct, but the distinction decreases when only viewing local information. But what is this perceptual distinction between satin and velvet based on?

In the rating tasks, participants were consistent in both conditions but less so in the crop condition. The agreement between participants varied depending on the perceptual attribute, which has been reported before (Fleming et al, 2013; Van Zuijlen et al, 2020).

Within the domain of computer vision, Schwartz and Nishino (2017) argued that visual material properties, such as shininess and hairiness, should be inherently local. Indeed, Geirhos, Rubisch, Michaelis, Bethge, Wichmann, and Brendel, (2019) showed that CNNs are strongly biased towards texture, that is, local image features. This implies that computer vision algorithms currently rely on local information. However, Geirhos et al. (2019) showed that CNNs trained to learn a shape-based representation (i.e., a bias for global information) improve on accuracy and robustness. Similarly, providing global and context information decreased the idiosyncrasy for the human data in our experiments. This implies that while both computer and human vision can form a clear or robust response from local information, the responses' robustness can be improved by providing global information.

The correlation matrices in Figure 5 showed that roughness was negatively correlated to shininess in both viewing conditions. This is in agreement with many reflectance distribution models such as for instance the microfacets model (Cook & Torrance, 1982), in which rough surfaces are modeled as a distribution of specular microfacets, which orientation distribution determines the surface roughness and resulting width of the reflectance lobe (the rougher, the less glossy, see also for instance Wendt & Faul, 2017; Honson, Huynh-Thu, Arnison, Monaghan, Isherwood, & Kim, 2020). We also see this negative correlation in the two PCA biplots (Figures 6 and 7).

**Figure 13.**
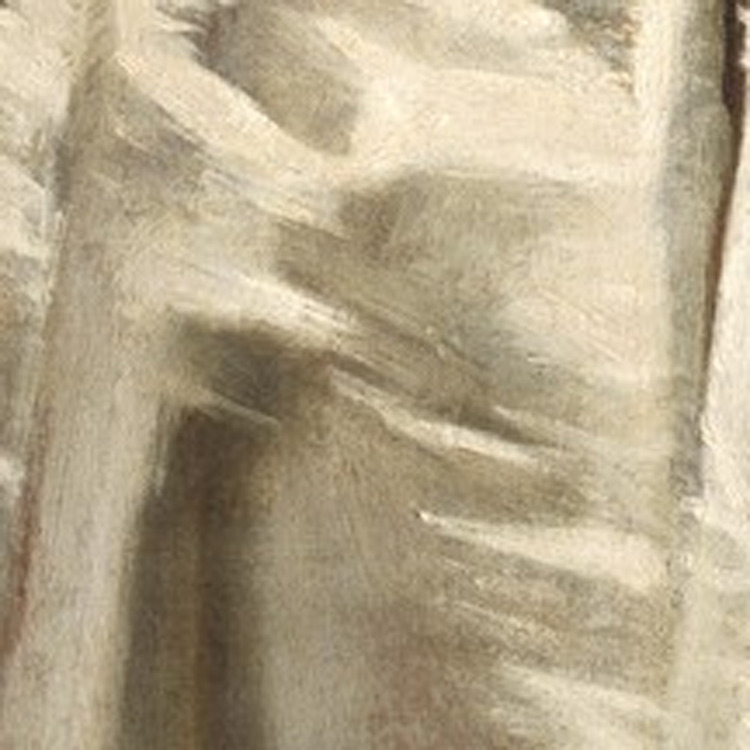
One crop that was identified as a possible outlier. With this stimulus included, a strong negative correlation was found between softness and roughness, which is surprising based on the literature. With this crop removed, the correlation is no longer significant. This might be due to the visibility of the individual brushstrokes, which gave rise to a perceptual ambiguity.

For roughness we found no correlation with softness for the full figure condition, which is in agreement with several studies that have shown that the main perceptual dimensions of tactile and visual perception of texture are roughness/smoothness and hardness/softness (Hollins, Faldowski, Rao, & Young, 1993; Okamoto, Nagano, & Yamada, 2013; Zhang et al., 2019). However, in the crop condition, we found a negative correlation between roughness and softness. This negative correlation might be ascribed to one outlier: a crop with clearly visible rough brushstrokes (see Figure 13), which was on average perceived to be the second roughest fabric and the least soft. Indeed, removing this crop from the data made the correlation no longer significant. Possibly the roughness of the brushstrokes for this specific stimulus introduced an element of ambiguity in the judgment of the surface roughness of the fabric.

Heaviness was significantly negatively correlated with shininess in both viewing conditions. In the crop condition, no size information was available. If participants were able to retrieve the material identity, heaviness could have been inferred through an “associative approach” (Schmidt et al., 2017). One possible association could have been that darker objects are perceived to be heavier than brighter ones (Walker, Scallon, & Francis, 2017; Vicovaro, Ruta, & Vidotto, 2019). From additional analysis, we found that ratings of heaviness in the crop condition were indeed highly negatively correlated with the mean luminance of the stimuli (*r* = −0.73, *p* < 0.001). Shininess, on the other hand, was highly and positively correlated with the mean luminance (*r* = 0.75, *p* < 0.001).

Softness, a material property relying on haptic information, is physically independent from the visual property of glossiness. However, they can be perceptually related since a perceptual association can be learned when intentionally induced (Ernst, 2007; Wismeijer, Gegenfurtner, & Drewing, 2012), or from prior experience, since glossy materials tend to be hard (Ingvarsdóttir & Balkenius, 2020). In the full figure condition, there was indeed a high and significant negative correlation between shininess and softness, likely due to the identification of the objects and of the materials they were made of. Paulun, Schmidt, Van Assen, and Fleming (2017) showed that the optical appearance of familiar materials creates expectations and influence stiffness perception. This might explain the lack of correlation between softness and shininess in the crop condition, where participants knew they were judging fabrics, but they were missing contextual information to recognize the fabrics’ material and thus were unable to draw from expectations.

In Figure 4 (top), we reported the attributes that were perceived to be significantly different between the two viewing conditions, per material. If we considered a single fabric, we observed additional variations of attributes between conditions (see Figure 8). This raised the question whether such variation in perception was due to our choice of the area to crop in the fabrics. Thus, in Experiment 2 we tested the relationship between the perception of shininess and softness and different areas cropped within the same fabric, spanning the whole fabric as much as possible. If different perceptions were triggered, they might be the result of the presence or absence of local image features within the crop. On the other hand, if all crops were perceived similarly, we might argue that local image features tend to be stable across the entire surface of the materials, at least within our set of stimuli.

The consistency within participants fluctuated greatly for different sets of crops, from 0.16 to 0.81 and from 0.18 to 0.77, for shininess and softness respectively. The consistency between participants showed similar fluctuations, from 0.13 to 0.92 for shininess, and from 0.08 to 0.78 for softness. The high agreement found for some sets of crops indicates that these crops evoked a clear and consistent perception. Simultaneously, the low agreement on other sets showed the opposite, namely that these crops were perceptually ambiguous. In the first experiment, stimuli presented with context and shape information, evoked a more consistent perception. It appears that cropping stimuli reduces the uniqueness of the evoked perception in some, but not all stimuli. The size, aspect ratio and area relative to the original image was kept constant within each set of crops, and can thus not explain the differences found. The local content of the crops within sets of crops must have caused the variety: the presence (or absence) of local image features in the crops of each set might be (in)sufficient to elicit a clear, consistent perception.

The results from the ANOVAs showed that crops were perceived to vary significantly in shininess within most of the crop sets. The presence of highlights on a surface is a well-known image feature for the perception of glossiness. According to Beck and Prazdny (1981), glossiness perception depends on the local presence of highlights, meaning that the direct area surrounding the highlight is perceived to be glossy, but not the whole surface per se. That is, they argue that glossiness perception is the direct response to local visual information, and not the result of some perceptual inference about the reflectance properties of the whole surface. Similar results are discussed by Berzhanskaya, Swaminathan, Beck, and Mingolla (2005). They found that perceived gloss decreases as a function of the distance from the highlight. Thus when different parts of an object are considered, gloss perception will differ among the different parts depending on their vicinity to the highlights. This local quality of glossiness is in agreement with our results on the perception of shininess differing between the crops of a fabric. We used three image features (mean luminance, coverage of the highlight and contrast of the highlight) to further analyze this relationship between the local image content and the evoked perception. In Figure 12 (top), we showed that the mean luminance of the crops was highly and positively correlated with almost all the crop sets for shininess. This finding is in line with Wiebel, Toscani, and Gegenfurtner (2015), who found that the mean luminance of photographs of real materials was a high-performance predictor, followed by the standard deviation of luminance, to differentiate between glossy and matte materials. Highlights are high-luminance regions of the surface, explaining the high correlation we observed between the mean luminance of the crops and the perceived shininess. Coverage of the highlights was also highly correlated with the perceived shininess for most of the crop sets. Coverage of highlights has been shown to be strongly associated with glossiness perception (Marlow, Kim, & Anderson, 2012; Marlow & Anderson, 2013), especially when coverage is the most reliable cue for the judgement of glossiness. This happens with objects whose shapes create higher variability in highlights’ coverage rather than contrast or sharpness, under the same illumination. For our stimuli, within the same fabric, the folding configuration caused high variations of coverage that we found to be related to significant variations in shininess perception between the different crops of a fabric. High highlights’ coverage is also related to higher mean luminance, given that the area of the surface covered with highlights, that is, the high-luminance regions, increases. We indeed found the correlation between the mean luminance and the coverage averaged over all the crop sets, to be high and significant (*r* = 0.78 *p* < 0.001). The third image feature that we measured, the highlights’ contrast, overall, was not strongly correlated with perceived shininess. In the three cases in which high and significant positive correlations were found, the contrast was also positively correlated with coverage. The opposite occurred for the only crop set that showed a significant negative correlation between contrast and shininess, that is, the high-contrast highlights covered the smallest regions of the fabrics’ surface.

For softness perception, the ANOVAs showed no significant differences for most of the crop sets. So, although the perception of shininess might depend on local image features, this might not hold for softness. A possible explanation for this finding could be that softness is a mechanical property, rather than an optical property and therefore less associated to the image features. Another related possibility is that the image features that were analyzed are simply not the key triggers for softness. The question then arises whether other local features might explain the data or whether mechanical attributes such as softness requires global features to explain the judgments.

**Figure 14.**
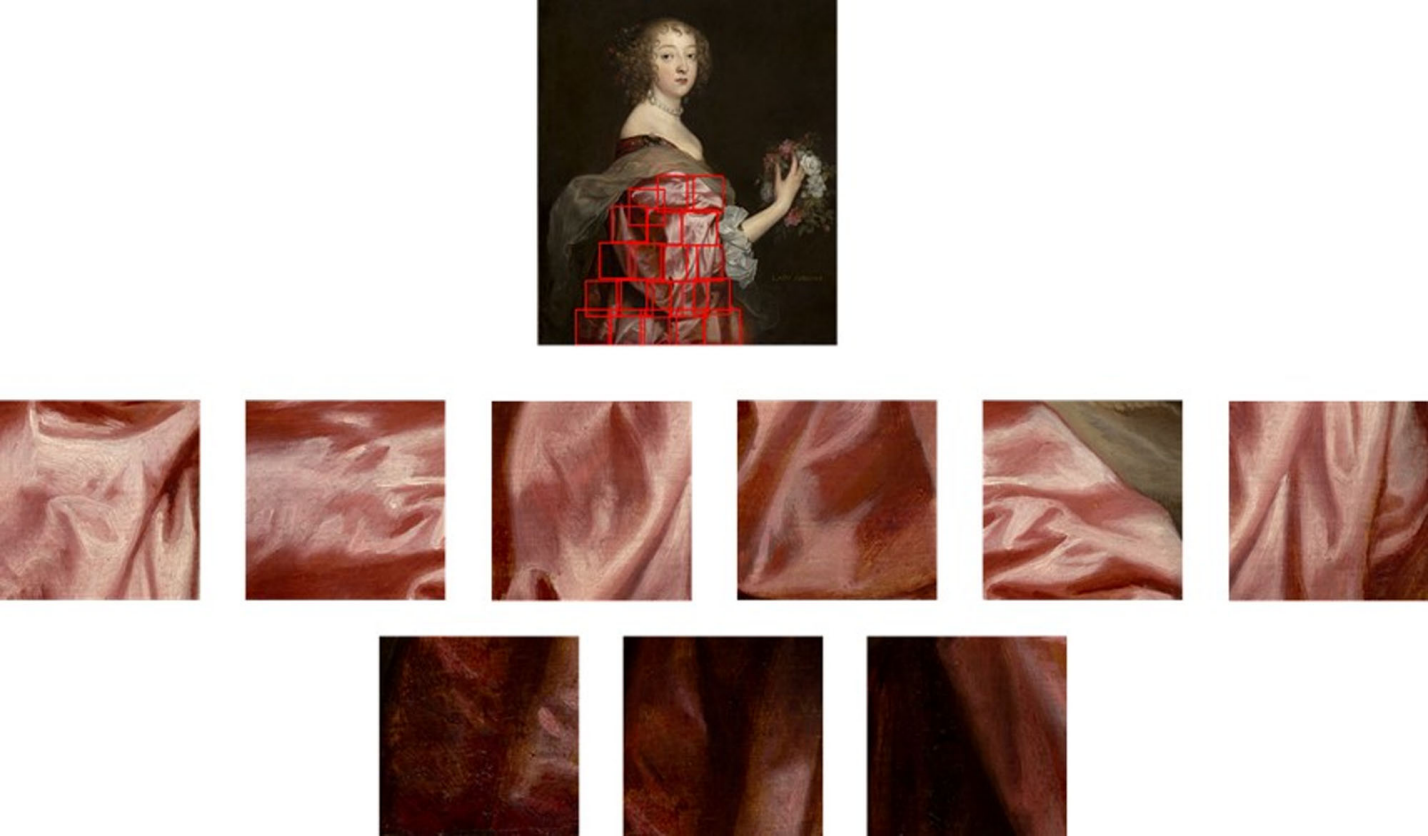
Visualization of the crop set 2 from the bottom of Figure 12. The image on top shows the locations where the crops were taken from the whole fabric. The crop in the top row were perceived to be significantly softer than the crops in the bottom row. Anthony van Dyck, *Catherine Howard, Lady d'Aubigny*, 1638, National Gallery of Art.

In the bottom section of Figure 12 we reported the three crop sets that were significantly different for softness perception. They all showed a high and significant positive correlation with the mean luminance of the crops. Two sets were also significantly positively correlated with the coverage of the highlights and one of these sets (crop set 2) is shown in Figure 14. The crops in the top row were perceived to be significantly softer that the crops in the bottom row. What is apparent from these two rows of crops is that in the top row, the high luminance and the high coverage of the highlights allow to clearly see the folding shape of the fabric, in contradistinction to those in the bottom row. Local shape features, like textiles’ folding, have been shown to play a role in the visual estimation of softness perception (Schmidt, Fleming & Valsecchi, 2020). For the stimuli shown in Figure 14, the visibility of the shape deformation because of the folding could have been the driving cue for the perception of different levels of softness between the crops. This is in agreement with Xiao et al. (2016), who showed that the three-dimensional folding configuration increases the accuracy of estimation of tactile material properties of fabrics.

Other cues, such as the brightened contours, might be related to visual perception of softness via a cognitive association with velvet (Paulun et al., 2017; Schmidt et al., 2017; Zhang et al., 2019). Further research is needed to understand how local and global information contribute to and possibly interact in material perception, and whether such mechanisms are dependent on the material and property under consideration.

**Figure 15.**
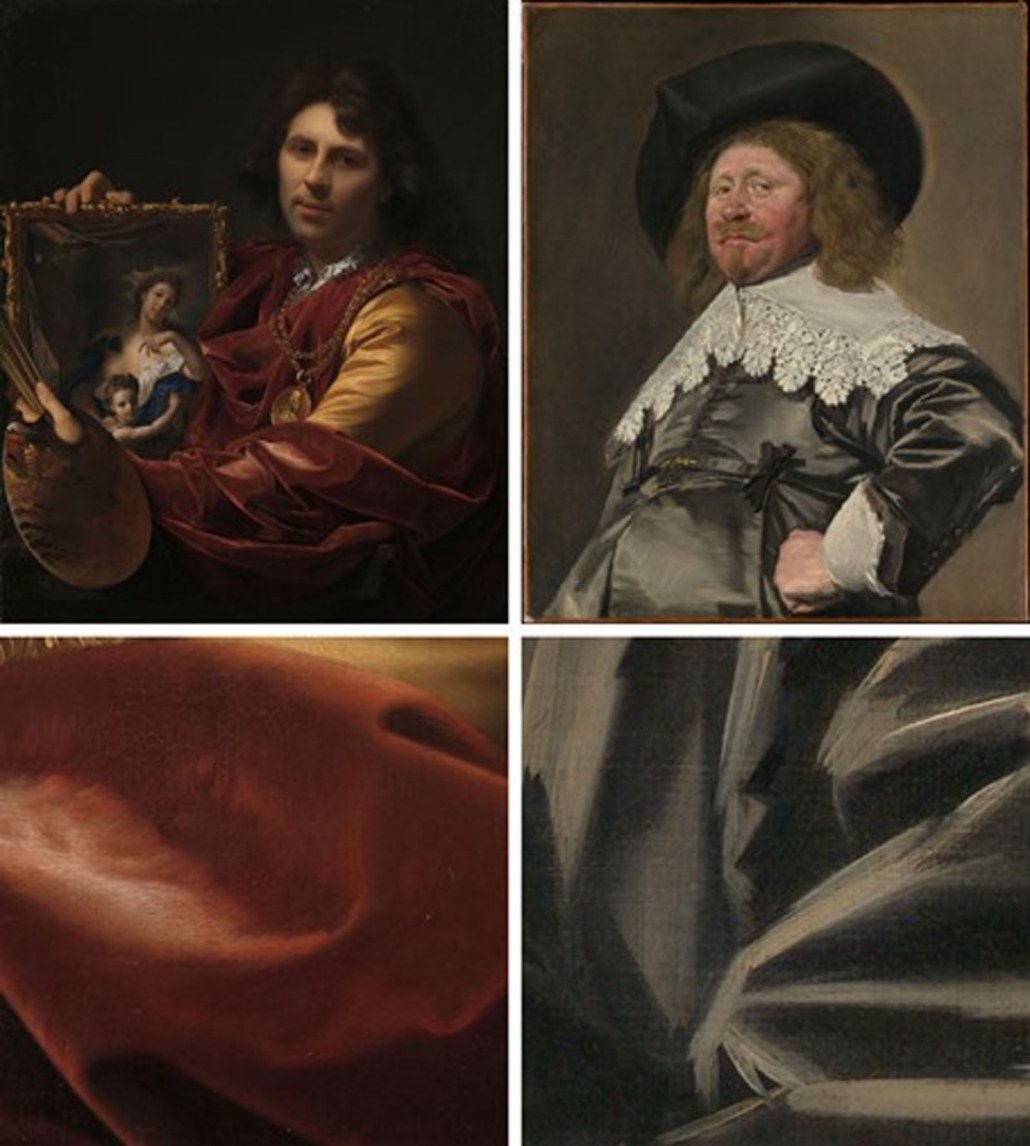
Examples from our stimuli set of a neat (left) and a loose use of brushstrokes (right), to illustrate the difference in these two styles which becomes apparent when moving closer to the physical painting—or when zooming in. The two crops (below) are from the crop-set that correspond to the paintings (above). Left: Adriaen van der Werff, *Self-portrait with the Portrait of his Wife, Margaretha van Rees, and their Daughter Maria,* 1699, Rijksmuseum. Right: Frans Hals, *Portrait of a Man, Possibly Nicolaes Pietersz Duyst van Voorhout, ca. 1636–38*, The Metropolitan Museum of Art*.*

Since the 15^th^ century, with the introduction of oil painting and a whole new range of possible visual effects, Netherlandish painters started to shift the attention from the rendering of space and volume to the rendering of materials, reaching their “golden age” in the 17^th^ century. When the separation between diffuse and specular illumination started to be acknowledged and exploited (Gombrich, 1976), painters could visually differentiate velvet from satin, instead of rendering all the fabrics equally matte. This novel use of the highlights is what we quantified in Experiment 2 via image analysis, and related to the perception of shininess and softness. However, there is a stylistic aspect of the paintings that we selected for our stimuli set, which we did not address here, in order to focus on the discussion on material perception. The paintings were made either with a neat, almost invisible brushwork (see Figure 15 left) or with loose brushstrokes (see Figure 15 right). These opposite pictorial manners were equally valued to produce a convincing effect (Gombrich, 1960), but their mechanisms are completely different. Paintings with the fine brushstrokes can be appreciated from a distance or from close by in a similar way, whereas paintings with coarse brushstrokes are unintelligible when one stands close or zooms in, but they make perfect sense and trigger a powerful convincing effect when seen in their entirety, at a proper distance.

The different brushworks might have introduced an additional source of noise in our data, but they also raised further questions, such as how is the pictorial style (fine vs coarse) related to the use of local and global image cues for material depiction and perception? Future work in this direction could contribute to the emerging field of art and perception.

## Conclusions

In this study, we found that warmth, heaviness, hairiness and softness are key attributes of the material signatures of velvet, whereas shininess is a key attribute of the signature of satin, when studying the depiction of both fabrics in 17^th^ century paintings. We further showed that the two fabrics, and their material signatures were clearly perceptually distinct when the stimuli were presented in the full figure condition. On the other hand, the cropped condition, depriving the visual system of object shape and context information, caused higher ambiguity and made the distributions for the measured perceptual attributes of the two materials less distinct.

In Experiment 2, we showed that the perceived shininess is not stable across one single fabric. The perception of the optical property shininess based on a cropped area of the fabric was shown to be correlated to the presence of diagnostic image features in the crop, namely highlights. Moreover, shininess perception increased with the coverage of the highlights and their mean luminance.

The haptic property of softness, instead, did not differ significantly between crops of the same fabric. Further analysis of the softness data suggested that perception of this haptic property might be driven by local and global shape cues.

In conclusion, we have shown that velvet and satin were depicted with distinct perceptual material signatures, which painters started to employ around the 15^th^ century, and highlights started to be exploited to render the characteristic appearance of different textiles (Gombrich, 1976). Highlights can be used to render the luster of satin and the softness of velvet by indicating not only how the fabric reflects light but also by revealing the shape of the folds. Local image features of the highlights were found to be sufficient to trigger significant variations in shininess perception, but not for softness. This indicates that shininess is a local material property, whereas softness might require more global visual information relating to shape.

## Supplementary Material

Supplement 1
