# Supplementary material for: Soft like velvet and shiny like satin: Perceptual material signatures of fabrics depicted in 17th century paintings
Source: J Vis. 2021 May 12;21(5):10. doi: 10.1167/jov.21.5.10 (PMC8132013; doi:10.1167/jov.21.5.10)

**Figure S1.** All the stimuli used in Experiment 1. For stimuli we display the full figure condition on the left, and the crop condition on the right. Note that both images are resized to fit the page while in the experiment stimuli were presented at 600x600 and 200x200 pixels for the full figure and crop condition, respectively. The first 11 are the satin stimuli, the remaining 8 are the velvet stimuli. All images reproduced here are are available under open access at a CC0 or CC BY 4.0 license. Two paintings (and the crops thereof) have not been reproduced here due to copy rights restraints.

- *Portrait of a Man, Possibly Nicolaes Pietersz Duyst van Voorhout*, by *Frans Hals*. 1637, The Metropolitan Museum of Art


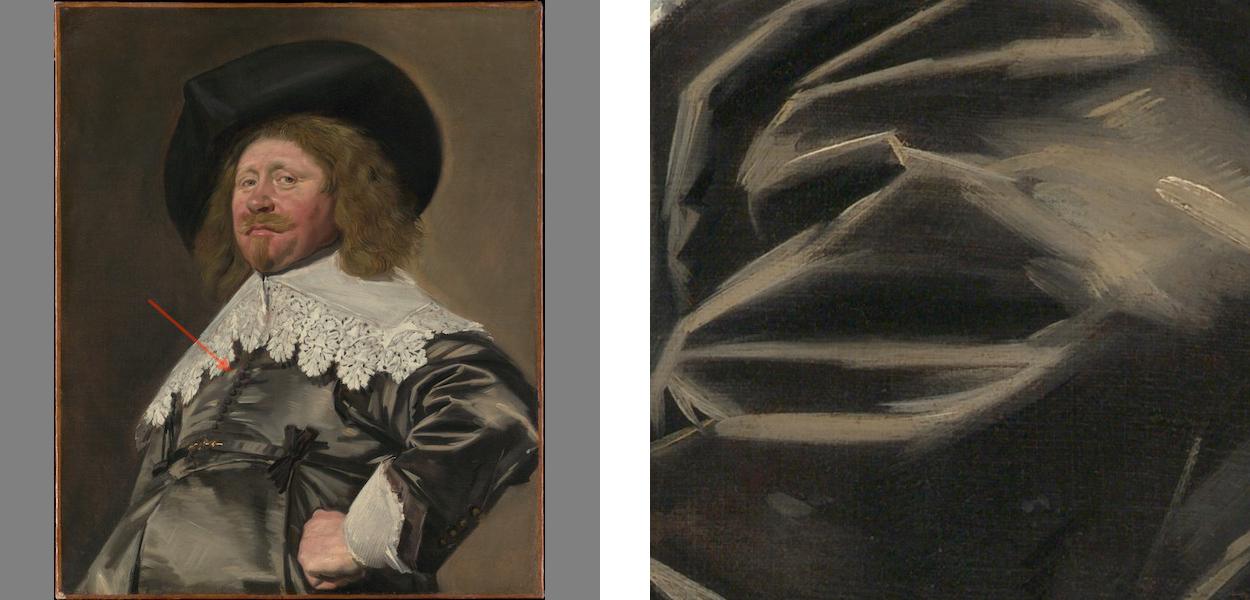


- *A Young Woman Composing a Piece of Music*, by *Gabriël Metsu*. 1664, Mauritshuis


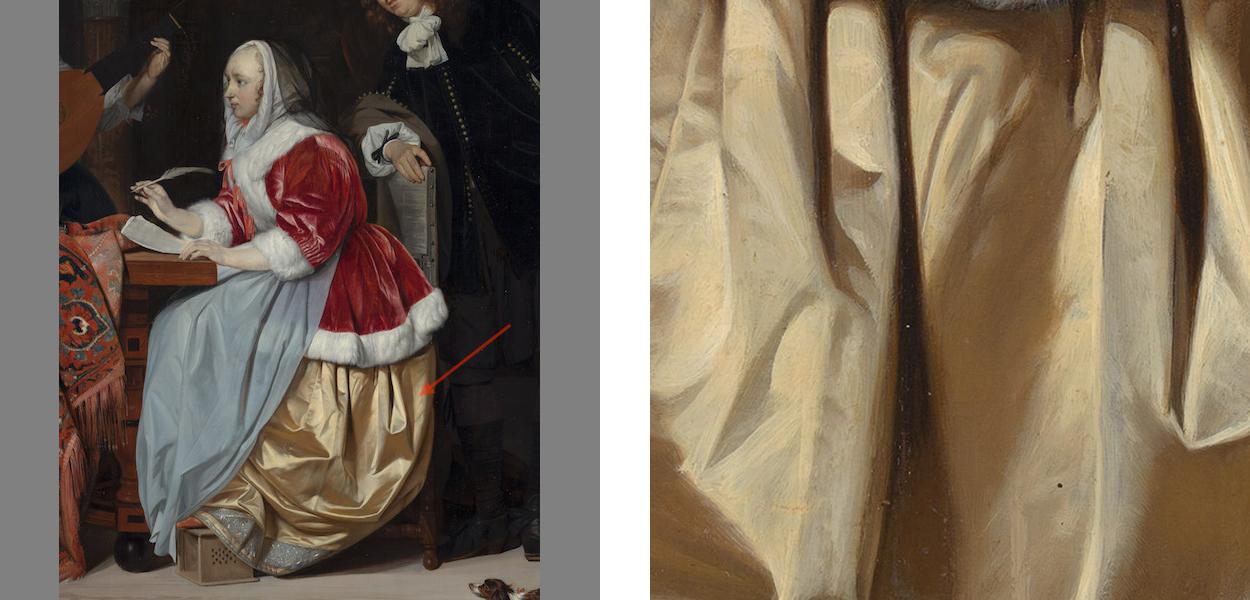


Stimuli continued the next page.

**Figure S1.** Continued

- *Portrait of a Man*, by Adriaen van der Werff. 1689, Mauritshuis


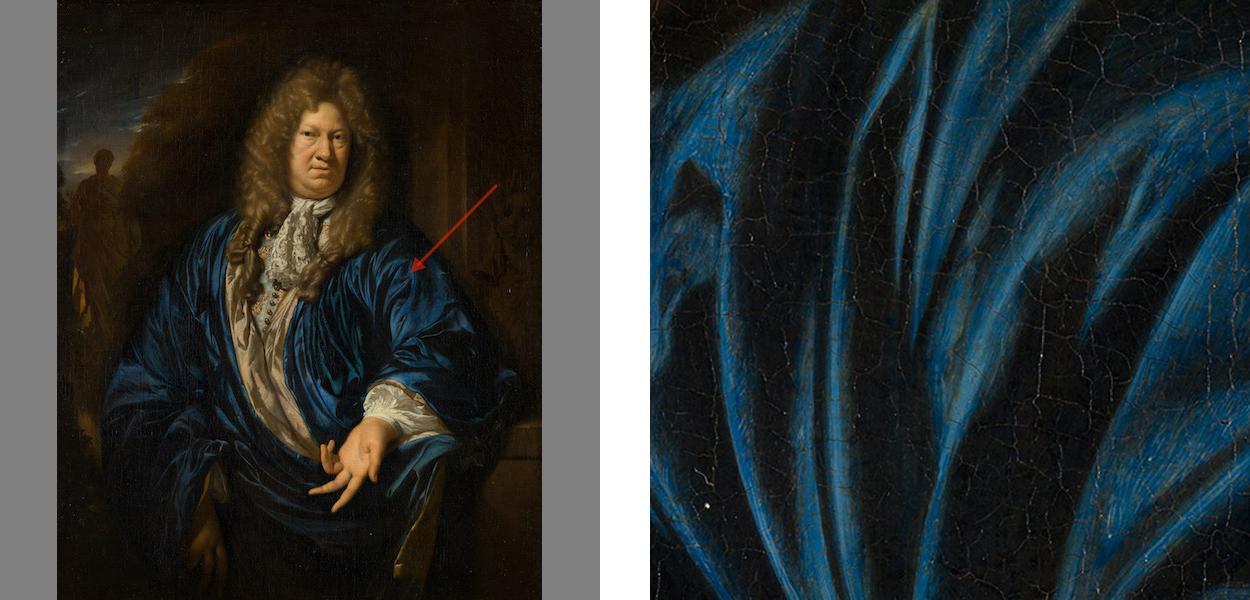


- *The Oyster Meal*, by *Frans van Mieris the Elder*. 1661, Mauritshuis


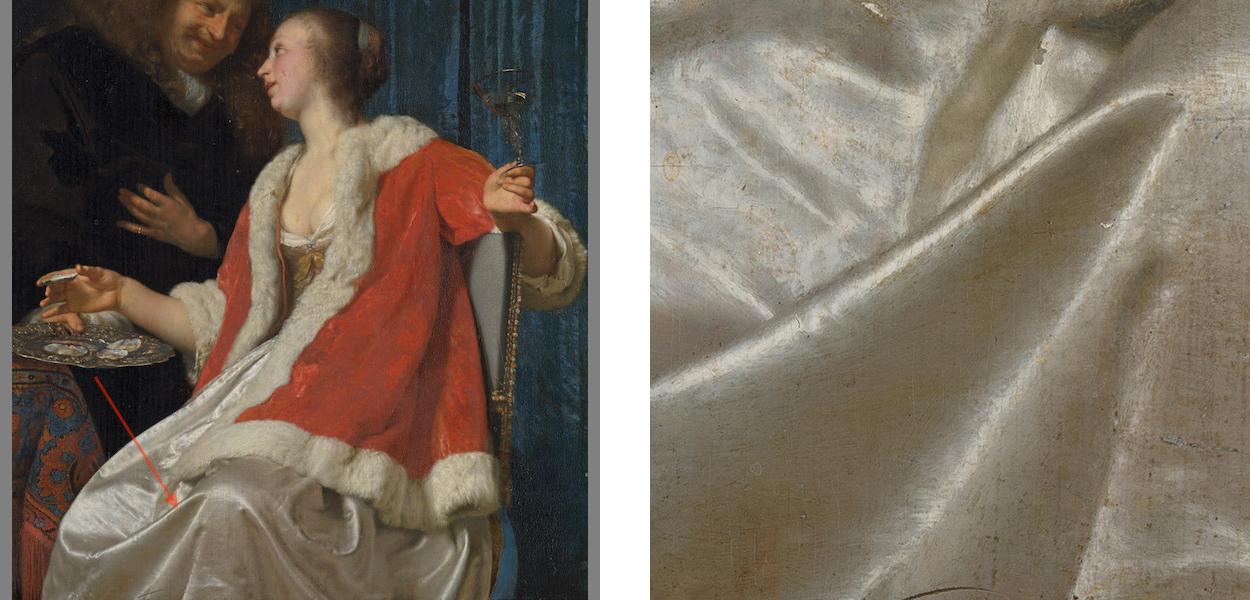


Stimuli continued the next page

**Figure S1.** Continued

- *Philip, Lord Wharton*, by *Anthony van Dyck*. 1632, National Gallery of Art


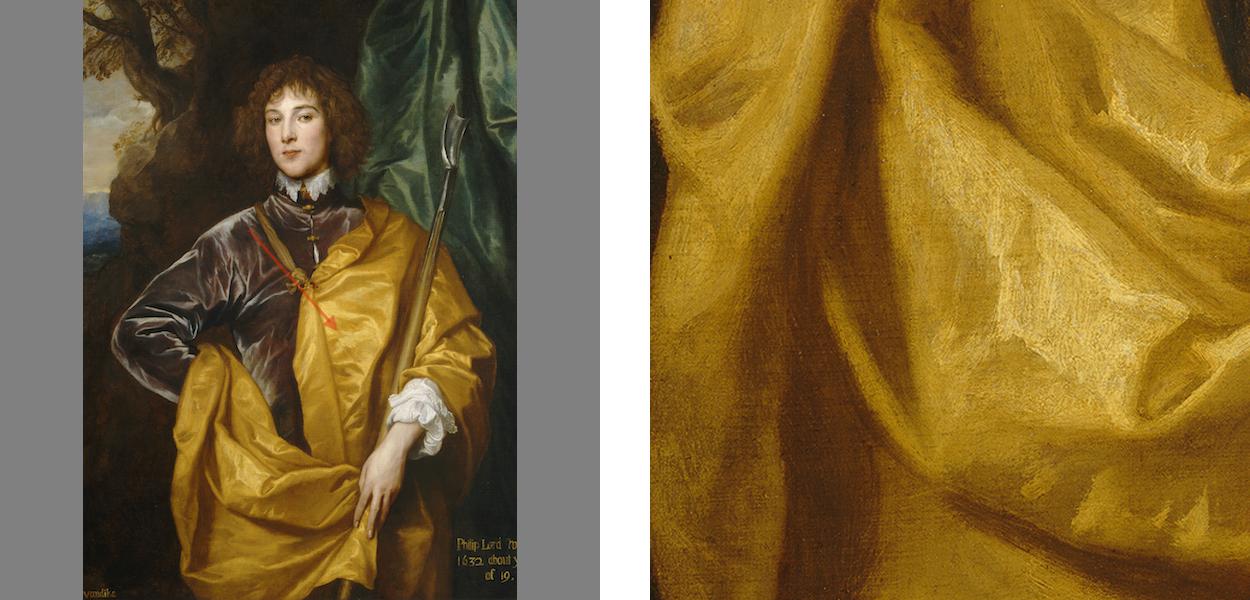


- *Catherine Howard, Lady d'Aubigny*, by *Anthony van Dyck*. 1638, National Gallery of Art


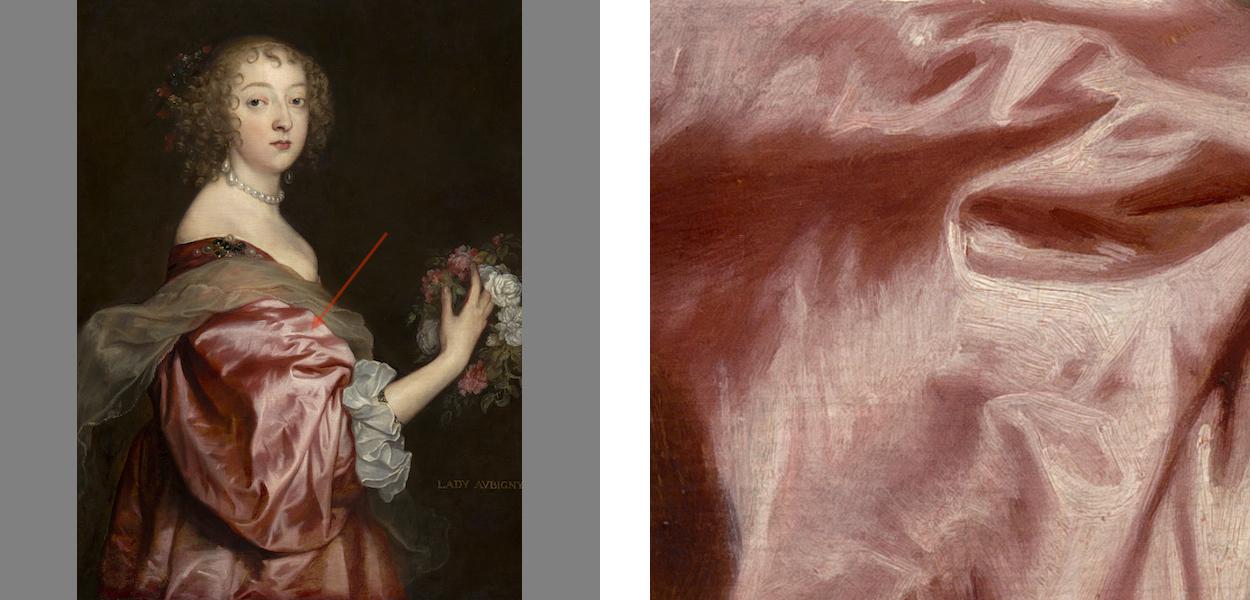


Stimuli continued the next page.

**Figure S1.** Continued

- *Marchesa Brigida Spinola Doria*, by *Peter Paul Rubens*. 1606, National Gallery of Art


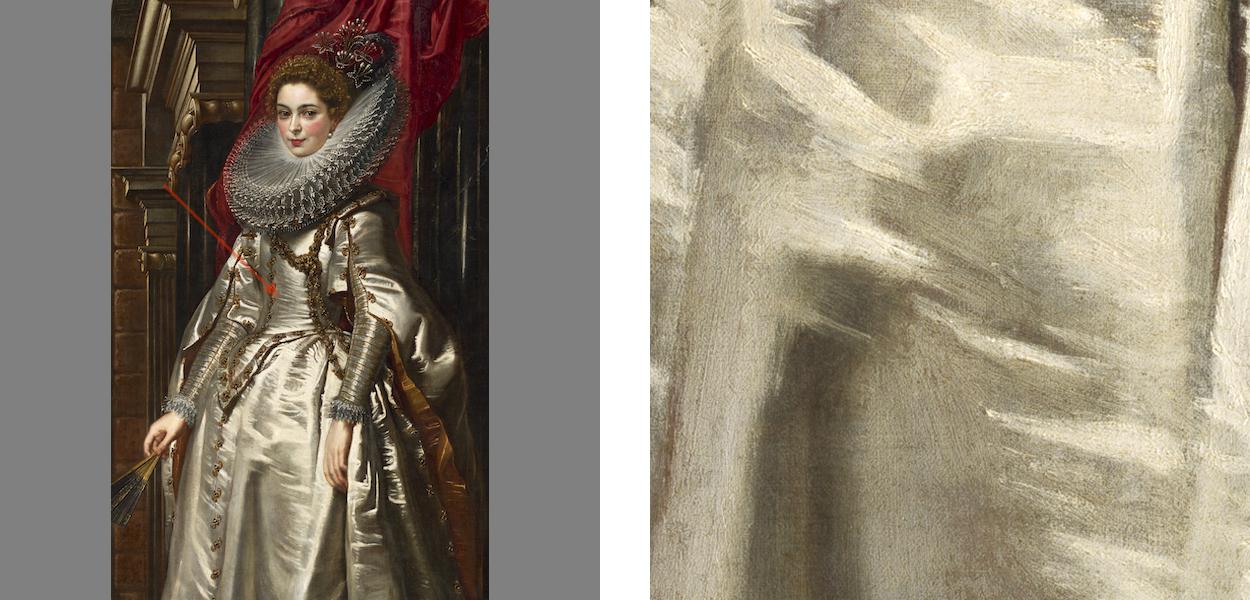


- *Queen Henrietta Maria with Sir Jeffrey Hudson*, by *Anthony van Dyck*. 1633, National Gallery of Art


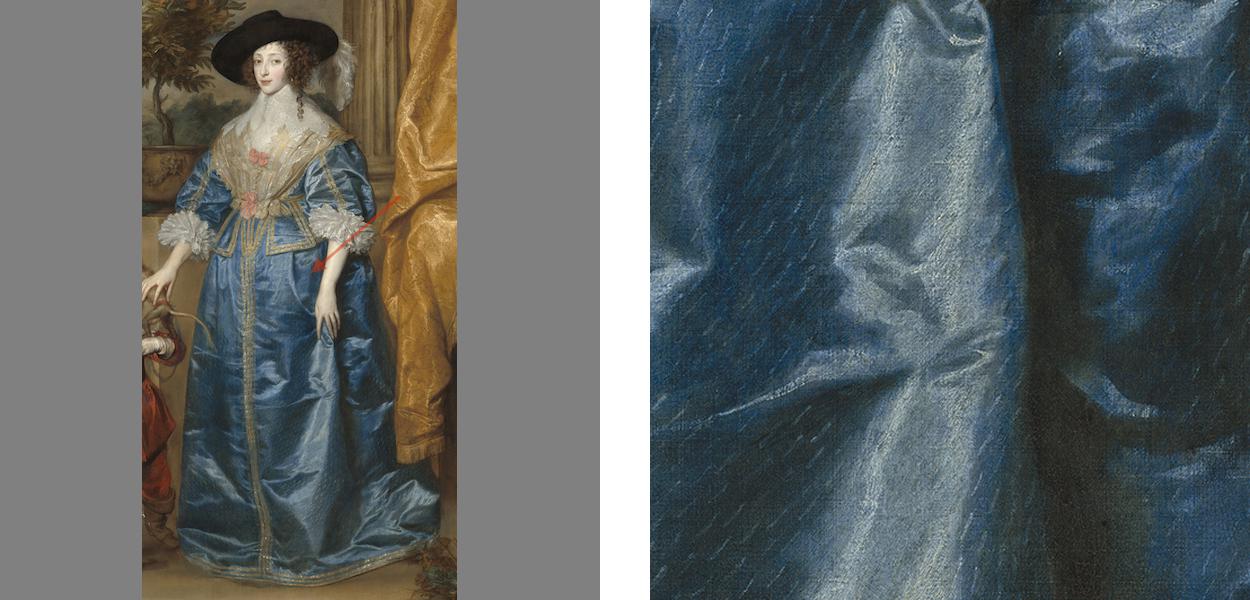


Stimuli continued the next page.

**Figure S1.** Continued

- *William II, Prince of Orange, and his Bride, Mary Stuart,* by *Anthony van Dyck.* 1641. The Rijksmuseum


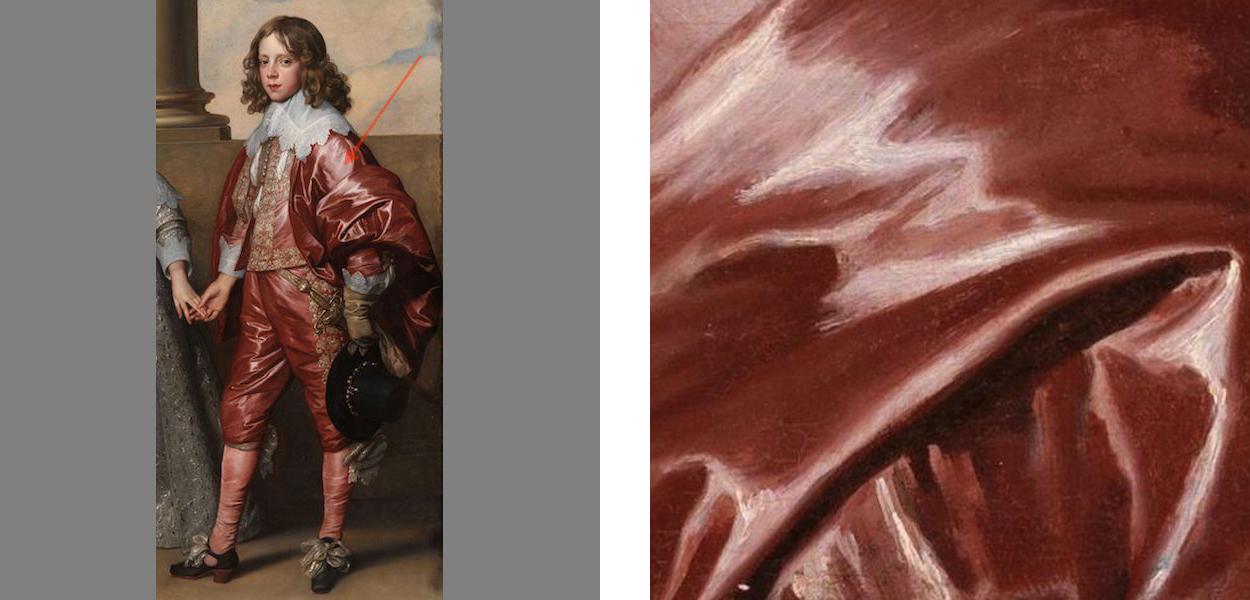


- *Pictura (An Allegory of Painting),* by *Frans van Mieris the Elder*. 1661, J. Paul Getty Museum


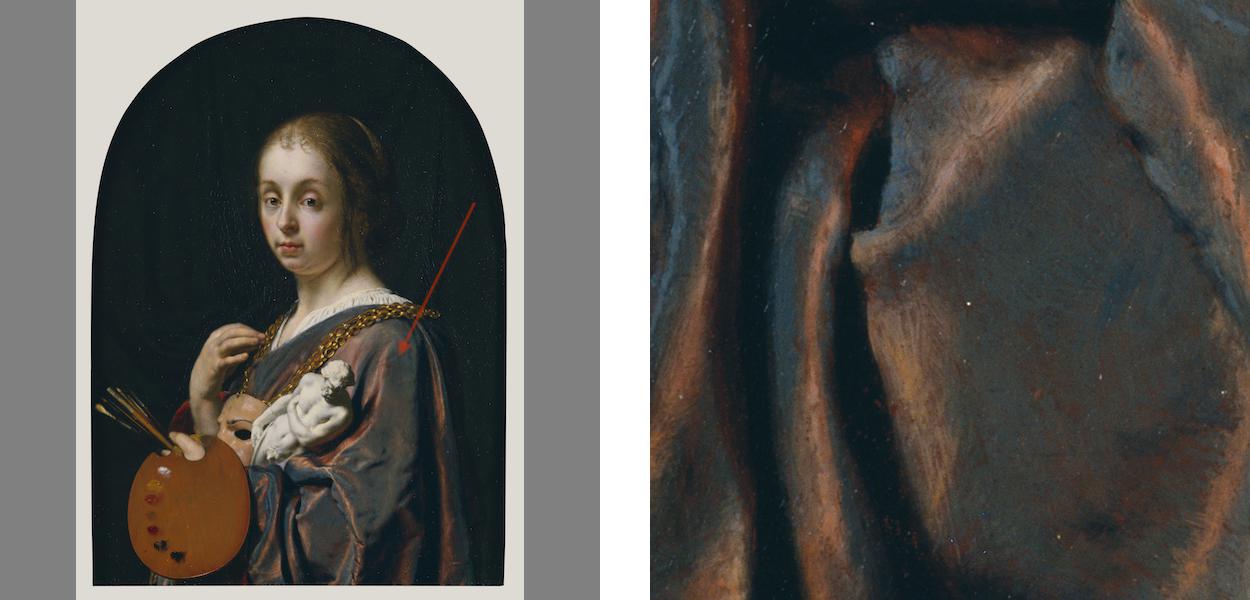


Stimuli continued the next page.

**Figure S1.** Continued

- *The Letter Writer*, by *Frans van Mieris (I)*. 1680, The Rijksmuseum


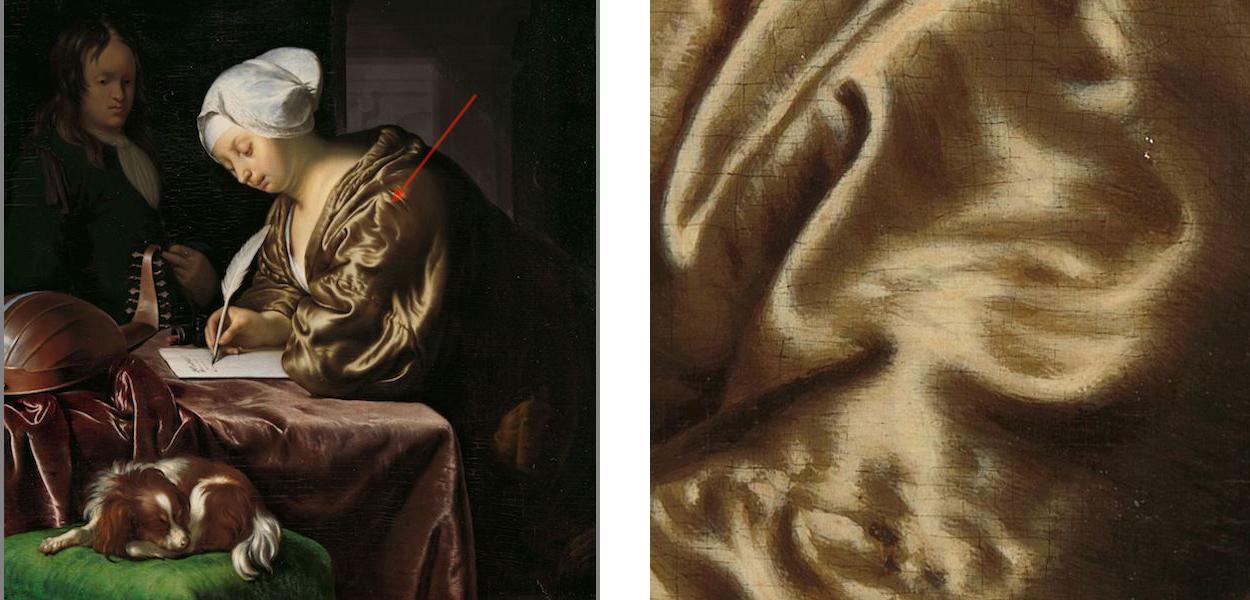


- *Portrait of Agostino Pallavicini*, by *Anthony van Dyck*. 1621, J. Paul Getty Museum


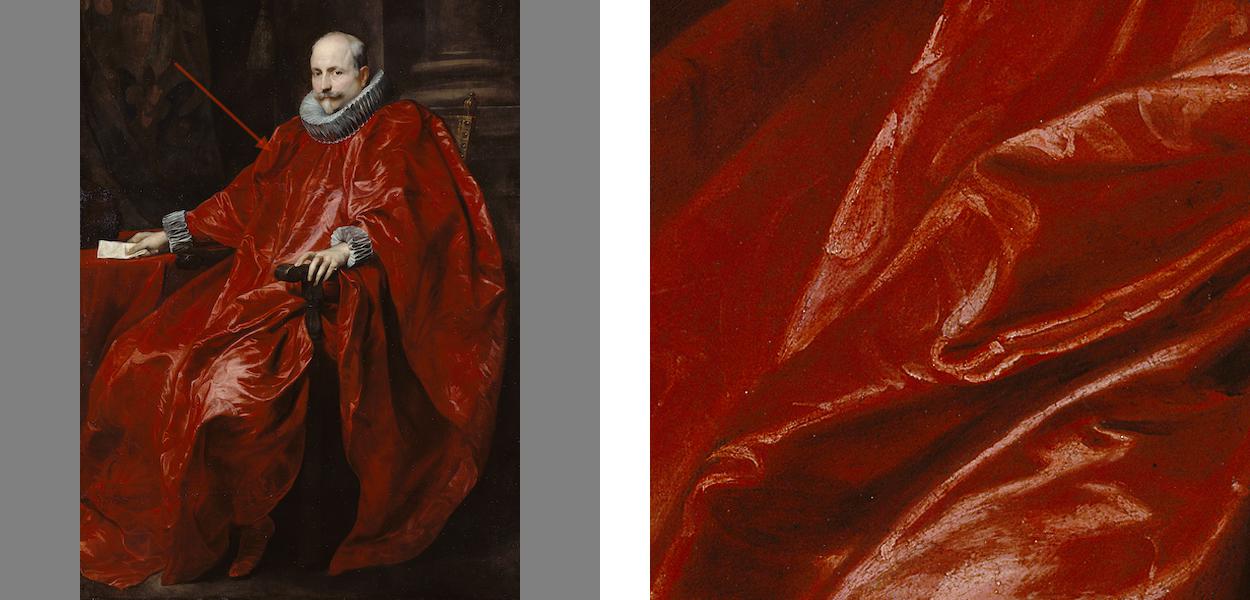


Stimuli continued the next page.

**Figure S1.** Continued

- *A Woman in a Red Jacket feeding a Parrot*, by *Frans van Mieris the Elder*. 1663, The National Gallery of London. Images not reproduced due to copy-rights restrictions.
- *The Letter Writer*, by *Frans van Mieris (I)*. 1680, The Rijksmuseum


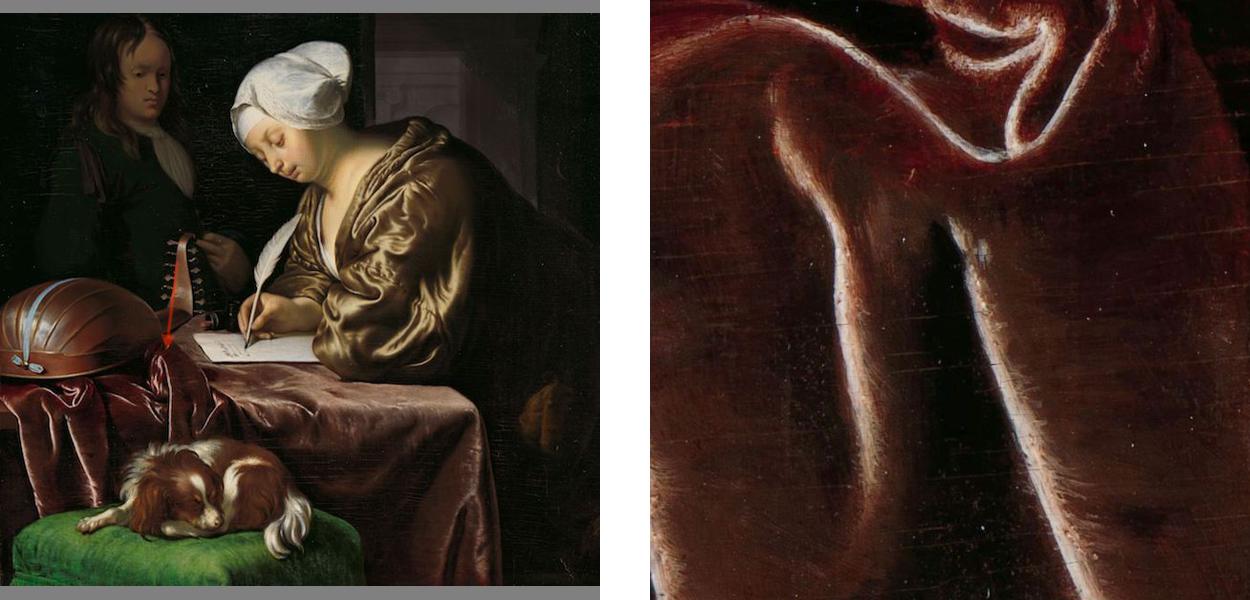


- *Old Woman Reading*, by *Gerard Dou*. 1631, The Rijksmuseum


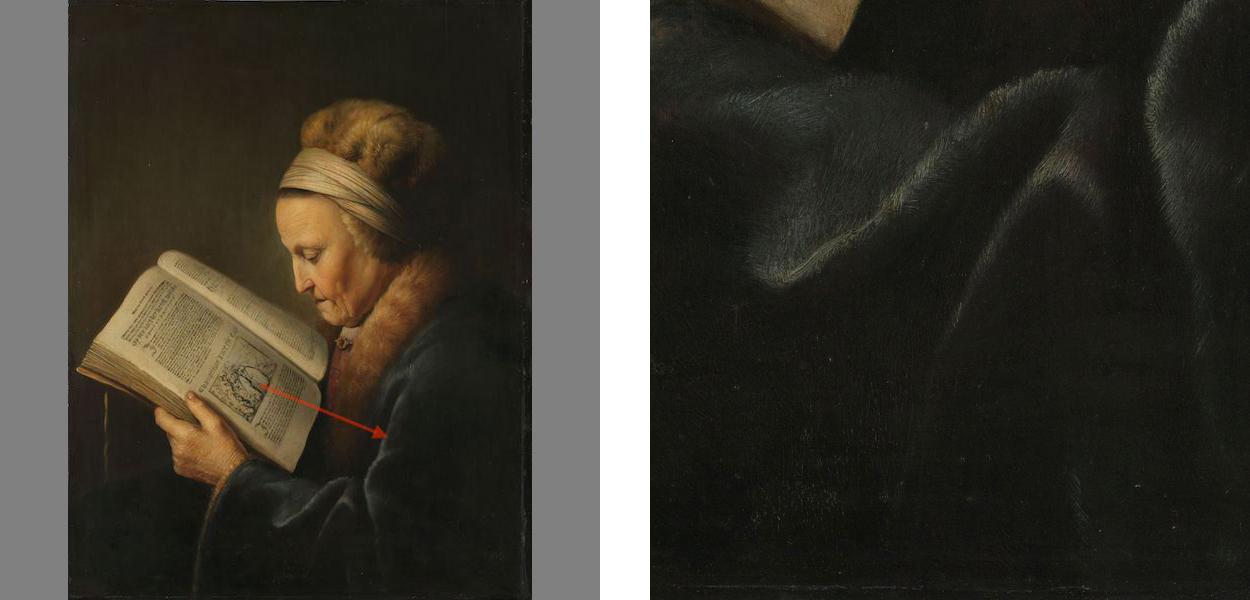


Stimuli continued the next page.

**Figure S1.** Continued

- *An Old Woman Reading, Probably the Prophetess Hannah*, by *Rembrandt van Rijn*. 1631, The Rijksmuseum


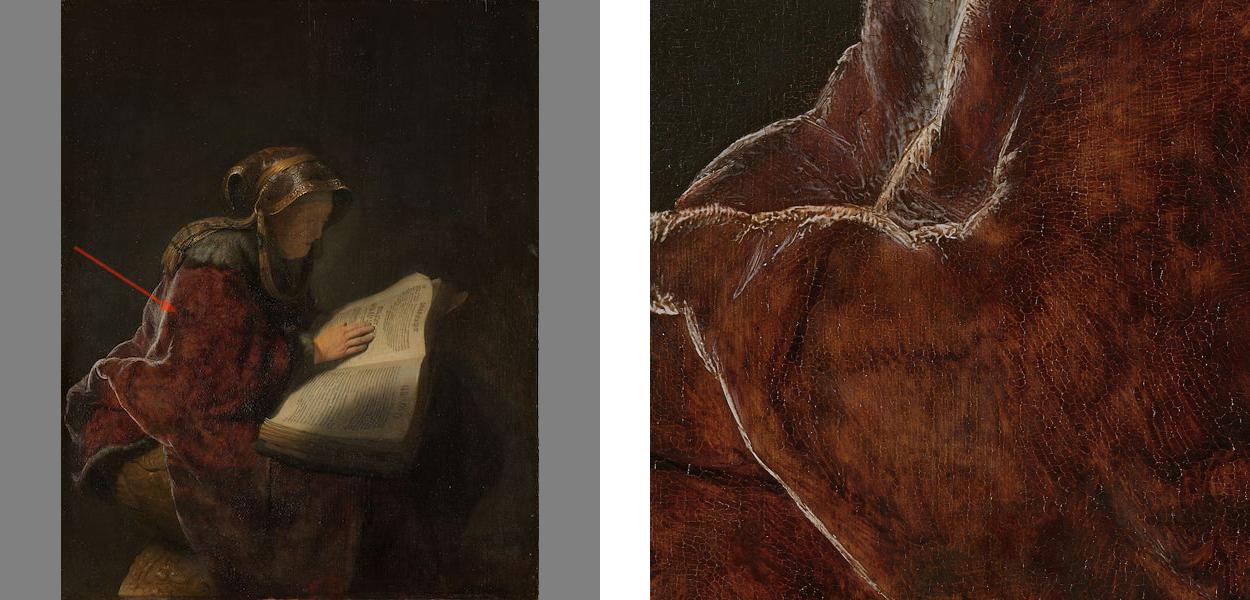


- *Self Portrait of the Artist, with a Cittern*, by *Frans van Mieris the Elder*. 1674, The National Gallery of London. Images not reproduced due to copy-rights restrictions.
- *Philip, Lord Wharton*, by *Anthony van Dyck*. 1632, National Gallery of Art


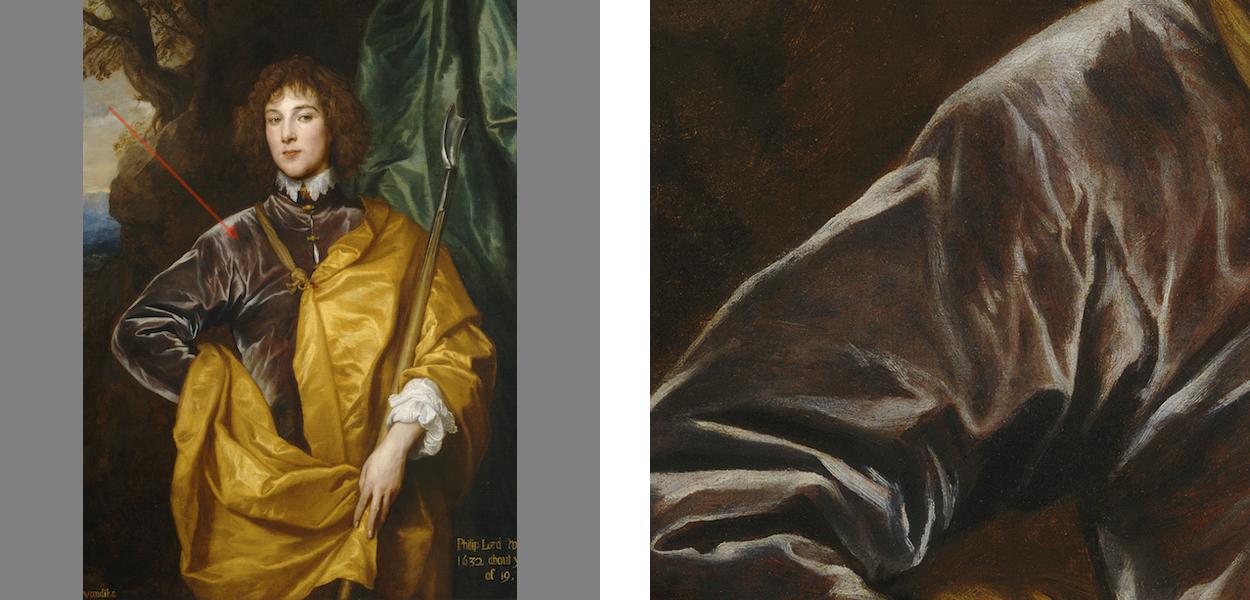


Stimuli continued the next page.

**Figure S1.** Continued

- *Self-portrait with the Portrait of his Wife*, *Margaretha van Rees, and their Daughter Maria,* by *Adriaen van der Werff*. 1699, The Rijksmuseum


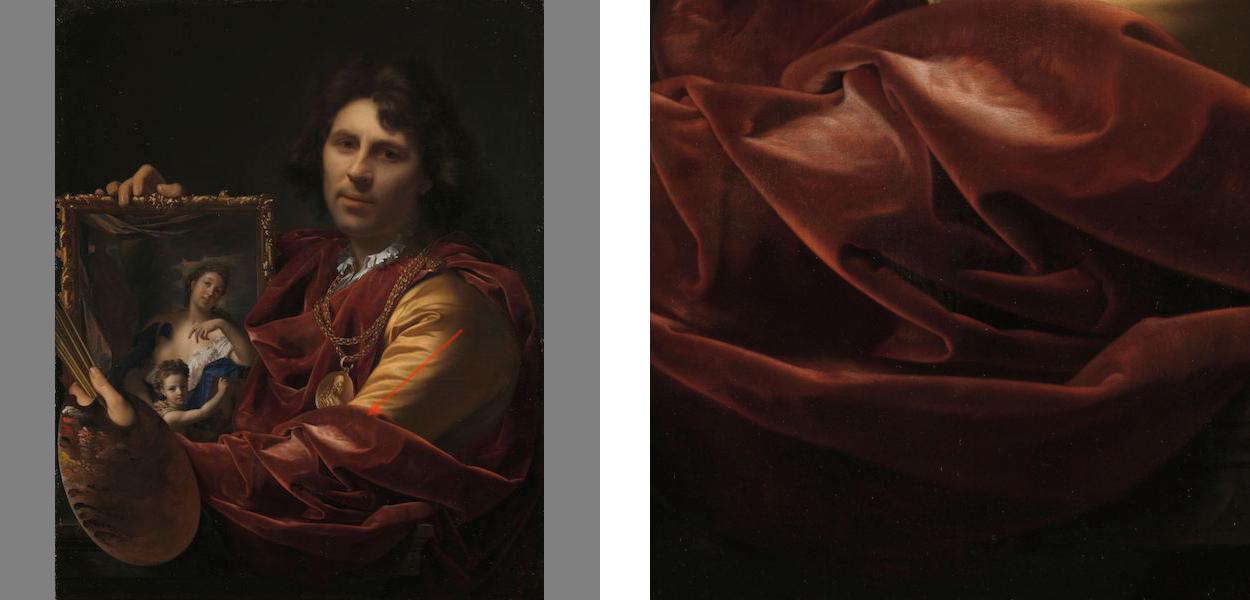


**Table S1.** ICC calculations for the inter-rater agreement in Experiment 1. The calculation was done using average rating, consistency agreement, two-way random effects model.

|  | ICC | 95% CI | | | F test with true value 0 | | | | | |
| --- | --- | --- | --- | --- | --- | --- | --- | --- | --- | --- |
|  |  | Lower Bound | Upper Bound | | Value | | *df1* | *df2* | | Sig. |
| *Full figure* |  |  | | |  | | | | | |
| Warmth | 0.78 | 0.61 | | 0.91 | 4.74 | | 18 | | 162 | <.001 |
| Hairiness | 0.92 | 0.85 | | 0.96 | 12.3 | 18 | | | 162 | <.001 |
| Softness | 0.75 | 0.54 | | 0.89 | 3.97 | 18 | | | 162 | <.001 |
| Heaviness | 0.89 | 0.79 | | 0.95 | 9.05 | 18 | | | 162 | <.001 |
| Shininess | 0.92 | 0.86 | | 0.96 | 12.8 | 18 | | | 162 | <.001 |
| Roughness | 0.61 | 0.27 | | 0.82 | 2.54 | 18 | | | 162 | <.01 |
|  |  |  | | |  | | | | | |
| *Crop* |  |  | | |  | | | | | |
| Warmth | 0.5 | 0.08 | | 0.77 | 1.99 | 18 | | | 162 | <.05 |
| Hairiness | -0.7 | -2.25 | | 0.21 | 0.56 | 18 | | | 162 | >.05 |
| Softness | 0.49 | 0.06 | | 0.77 | 1.97 | 18 | | | 162 | <.05 |
| Heaviness | 0.55 | 0.17 | | 0.79 | 2.22 | 18 | | | 162 | <.01 |
| Shininess | 0.71 | 0.47 | | 0.87 | 3.5 | 18 | | | 162 | <.001 |
| Roughness | 0.78 | 0.6 | | 0.9 | 4.63 | 18 | | | 162 | <.001 |

**Figure S2.** All the stimuli used in Experiment 2. The first 11 are the satin stimuli, the remaining 8 are the velvet stimuli.

- *Stimulus 1.* Crops taken from *Portrait of a Man, Possibly Nicolaes Pietersz Duyst van Voorhout*, by *Frans Hals*. 1637, The Metropolitan Museum of Art


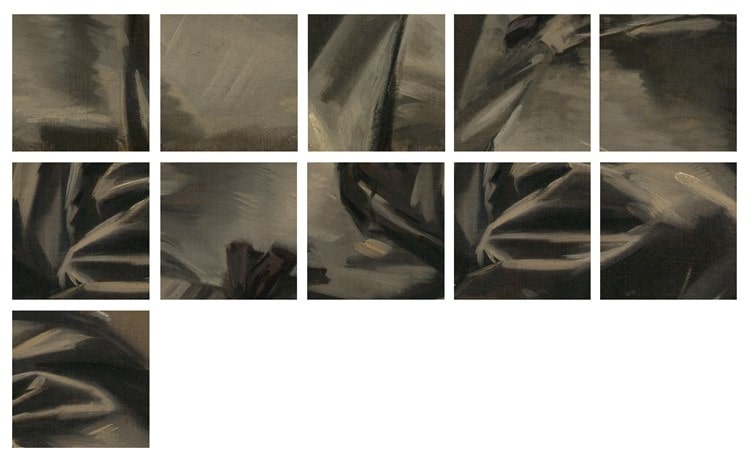


- *Stimulus 2.* Crops taken from *A Young Woman Composing a Piece of Music*, by *Gabriël Metsu*. 1664, Mauritshuis
  - Crop set 1 in Figure 12 shininess


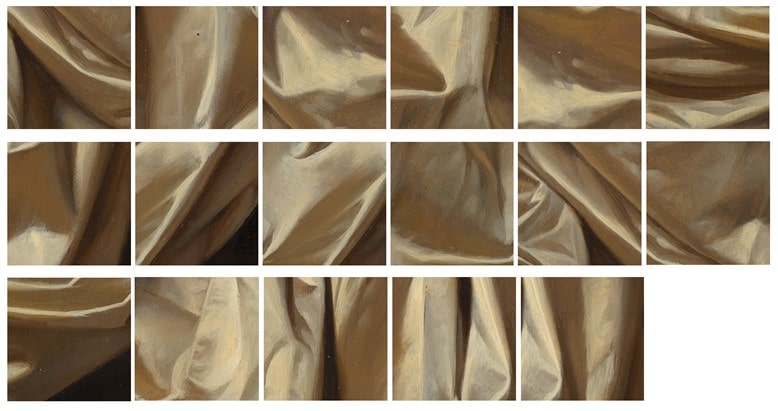


Stimuli continued the next page.

**Figure S2.** Continued

- *Stimulus 3.* Crops taken from *Portrait of a Man*, by *Adriaen van der Werff*. 1689, Mauritshuis
  - Crop set 2 in Figure 12 shininess


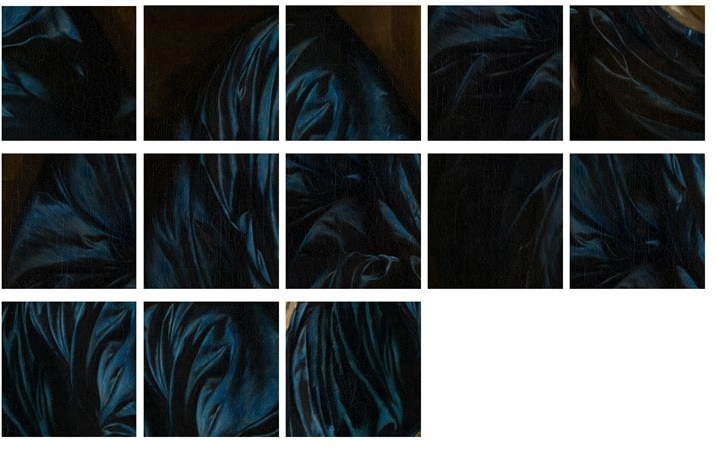


- *Stimulus 4.* Crops taken from *The Oyster Meal*, by *Frans van Mieris the Elder*. 1661, Mauritshuis
  - Crop set 3 in Figure 12 shinine


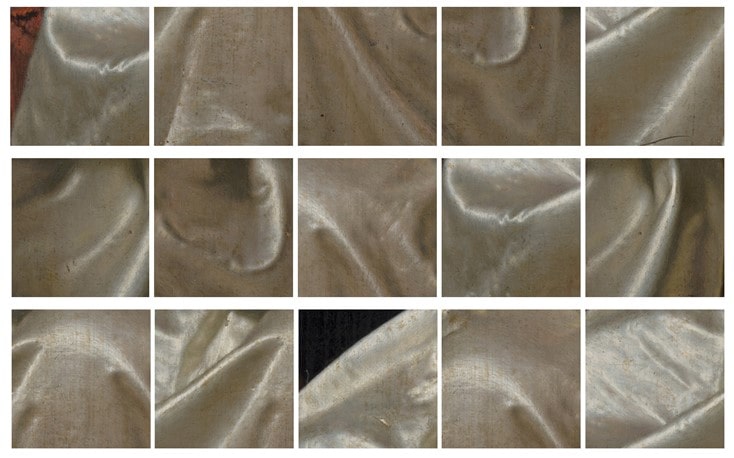


Stimuli continued the next page.

**Figure S2.** Continued

- *Stimulus 5.* Crops taken from *Philip, Lord Wharton*, by *Anthony van Dyck*. 1632, National Gallery of Art
  - Crop set 4 in Figure 12 shininess
  - Crop set 1 in Figure 12 softness


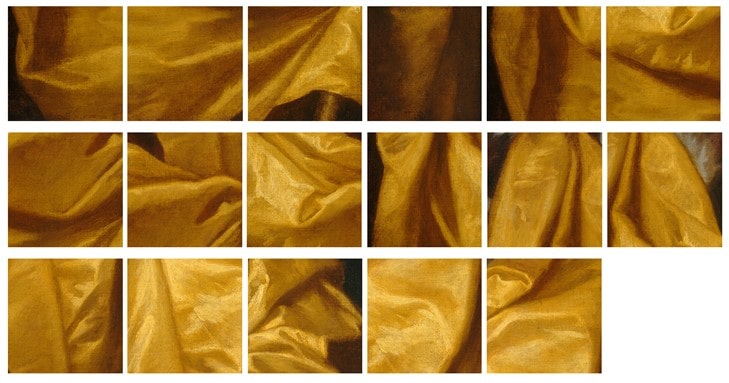


- *Stimulus 6.* Crops taken from *Catherine Howard, Lady d'Aubigny*, by *Anthony van Dyck*. 1638, National Gallery of Art
  - Crop set 5 in Figure 12 shininess
  - Crop set 2 in Figure 12 softness


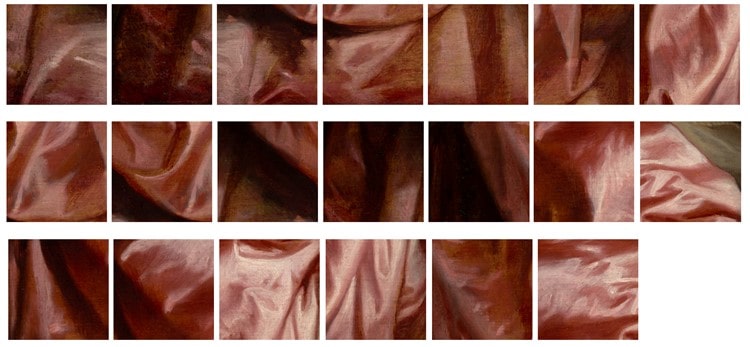


Stimuli continued the next page.

**Figure S2.** Continued

- *Stimulus 7.* Crops taken from *Marchesa Brigida Spinola Doria*, by *Peter Paul Rubens*. 1606, National Gallery of Art
  - Crop set 6 in Figure 12 shininess


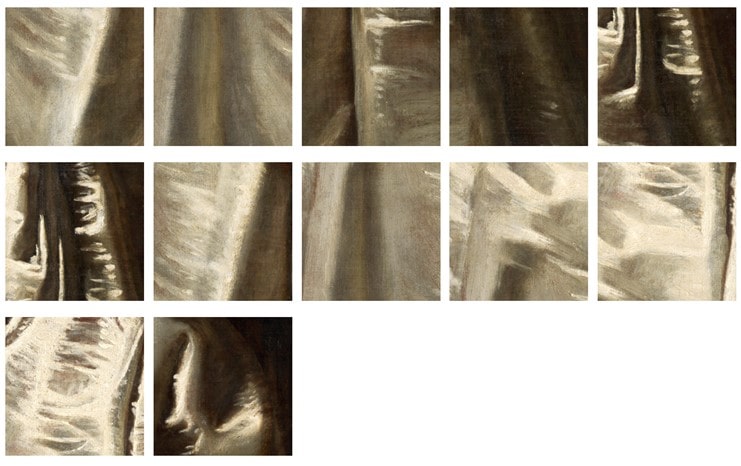


- *Stimulus 8.* Crops taken from *Queen Henrietta Maria with Sir Jeffrey Hudson*, by *Anthony van Dyck*. 1633, National Gallery of Art
  - Crop set 7 in Figure 12 shininess


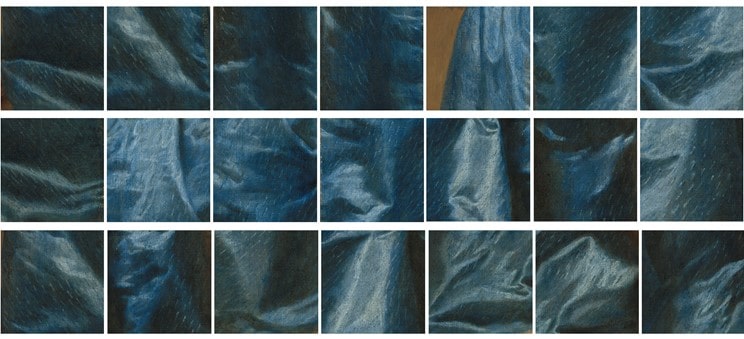


Stimuli continued the next page.

**Figure S2.** Continued

- *Stimulus 9.* Crops taken from *William II, Prince of Orange, and his Bride, Mary Stuart,* by *Anthony van Dyck.* 1641. The Rijksmuseum
  - Crop set 8 in Figure 12 shininess.


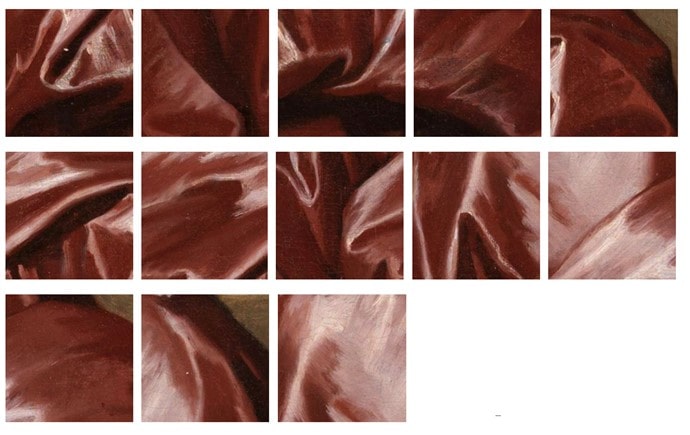


- *Stimulus 10.* Crops taken from *Pictura (An Allegory of Painting),* by *Frans van Mieris the Elder*. 1661, J. Paul Getty Museum
  - Crop set 9 in Figure 12 shininess.


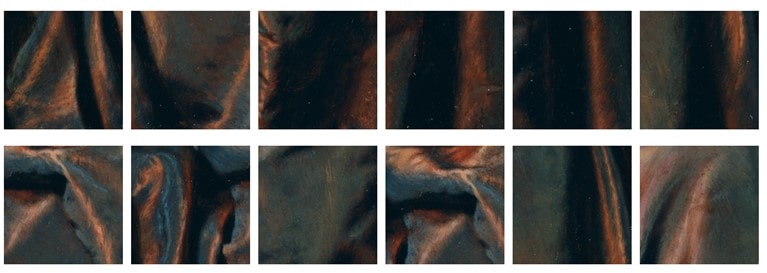


Stimuli continued the next page.

**Figure S2.** Continued

- *Stimulus 11* Crops taken from *The Letter Writer*, by *Frans van Mieris (I)*. 1680, The Rijksmuseum
  - Crop set 10 in Figure 12 shininess.


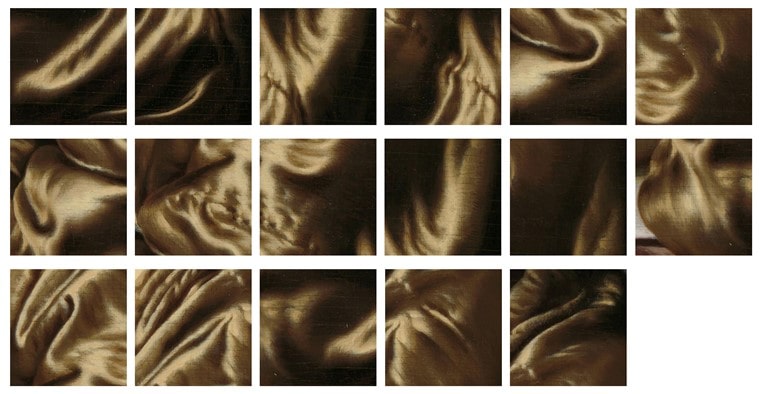


- *Stimulus 12.* Crops taken from *Portrait of Agostino Pallavicini*, by *Anthony van Dyck*. 1621, J. Paul Getty Museum
  - Crop set 11 in Figure 12 shininess.


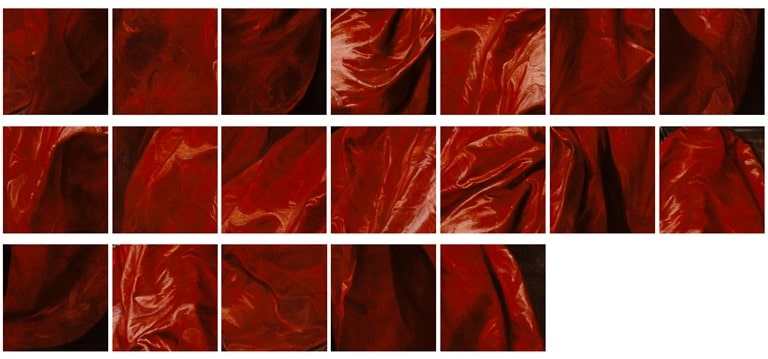


- *Stimulus 13. A Woman in a Red Jacket feeding a Parrot*, by *Frans van Mieris the Elder*. 1663, The National Gallery of London
  - Crop set 12 in Figure 12 shininess.
  - Images not reproduced due to copy-rights restrictions.

Stimuli continued the next page.

**Figure S2.** Continued

- *Stimulus 14.* Crops taken from *The Letter Writer*, by *Frans van Mieris (I)*. 1680, The Rijksmuseum
  - Crop set 13 in Figure 12 shininess.


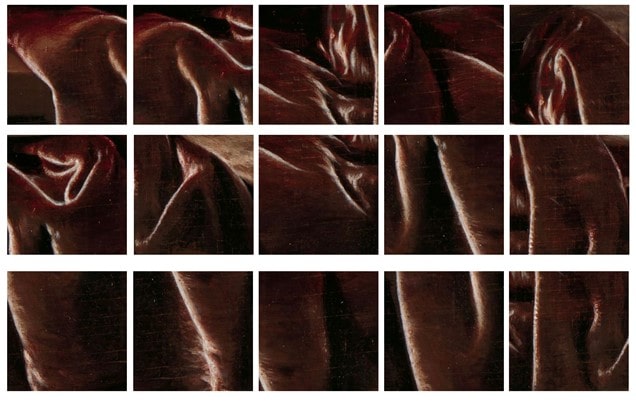


- *Stimulus 15.* Crops taken from *Old Woman Reading*, by *Gerard Dou*. 1631, The Rijksmuseum
  - Crop set 14 in Figure 12 shininess.


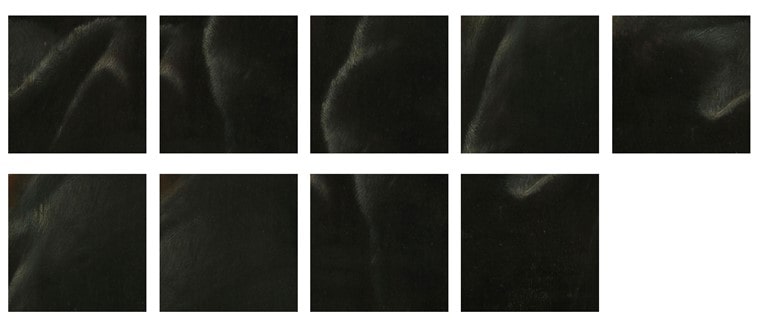


Stimuli continued the next page.

**Figure S2.** Continued

- *Stimulus 16.* Crops taken from *An Old Woman Reading, Probably the Prophetess Hannah*, by *Rembrandt van Rijn*. 1631, The Rijksmuseum
  - Crop set 15 in Figure 12 shininess.


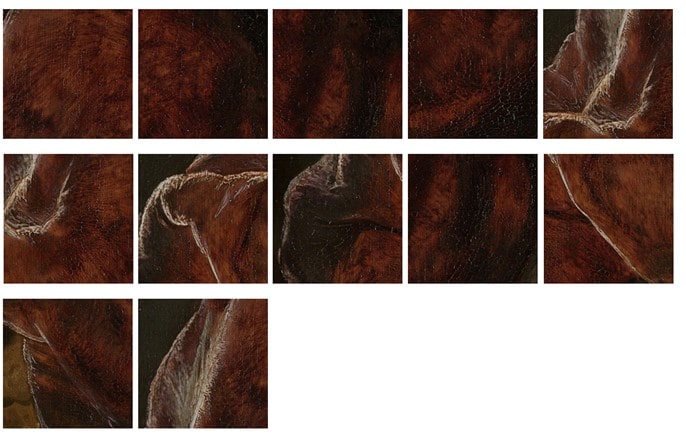


- *Stimulus 17.* Crops taken from *Self Portrait of the Artist, with a Cittern*, by *Frans van Mieris the Elder*. 1674, The National Gallery of London
  - Crop set 3 in Figure 12 softness.
  - Images not reproduced due to copy-rights restrictions.

Stimuli continued the next page.

**Figure S2.** Continued

- *Stimulus 18.* Crops taken from *Philip, Lord Wharton*, by *Anthony van Dyck*. 1632, National Gallery of Art
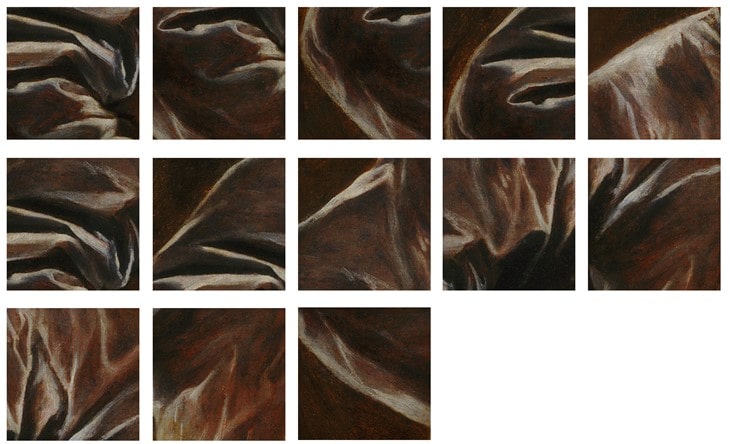

- *Stimulus 19.* Crops taken from *Self-portrait with the Portrait of his Wife*, *Margaretha van Rees, and their Daughter Maria,* by *Adriaen van der Werff*. 1699, The Rijksmuseum


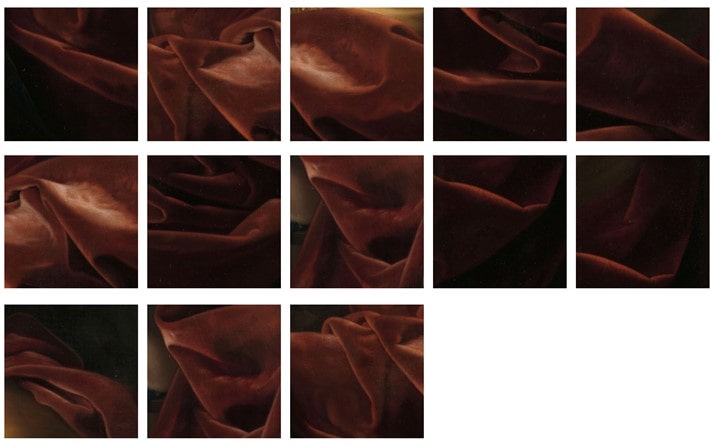

Supplement: Supplement 1 [file jovi-21-5-10_s001.docx]
